# Lycorine Derivative Inhibits SARS‐CoV‐2 Replication by Reducing −1 Programmed Ribosomal Frameshifting via Targeting ZAP

**DOI:** 10.1002/mco2.70715

**Published:** 2026-04-06

**Authors:** Tingfu Du, Ruixue Liu, Xintian Zhang, Longying Shen, Cong Tang, Junbin Wang, Yu Cheng, Wenhai Yu, Bin Yin, Shuaiyao Lu, Xiandao Pan, Xiaozhong Peng

**Affiliations:** ^1^ Institute of Medical Biology Chinese Academy of Medical Sciences & Peking Union Medical College Kunming China; ^2^ State Key Laboratory of Respiratory Health and Multimorbidity National Center of Technology Innovation For Animal Model Key Laboratory of Pathogen Infection Prevention and Control (Peking Union Medical College) Ministry of Education Institute of Laboratory Animal Science Chinese Academy of Medical Sciences & Peking Union Medical College Beijing China; ^3^ State Key Laboratory of Bioactive Substance and Function of Natural Medicines and Beijing Key Laboratory of Active Substances Discovery and Druggability Evaluation Institute of Materia Medica Chinese Academy of Medical Sciences & Peking Union Medical College Beijing China; ^4^ Department of Molecular Biology and Biochemistry Institute of Basic Medical Sciences Medical Primate Research Center Neuroscience Center Chinese Academy of Medical Sciences & Peking Union Medical College Beijing China

**Keywords:** –1 programmed ribosomal frameshifting, antiviral agent, lycorine derivatives, SARS‐CoV‐2, zinc‐finger antiviral protein

## Abstract

The ongoing evolution of SARS‐CoV‐2 and its immune‐evading variants underscores an urgent requirement for broad‐spectrum antiviral drugs. In this study, a series of lycorine derivatives was synthesized. This led to the identification of compound **7** as a promising antiviral candidate. Compound **7** exhibited potent inhibitory activity against SARS‐CoV‐2 and its variants, including Alpha, Beta, Delta, and Omicron, in vitro. The antiviral efficacy of compound **7** was then validated in vivo. Treatment with compound **7** significantly reduced viral loads and alleviated lung pathologies in SARS‐CoV‐2‐infected hamsters. Mechanistically, compound **7** directly targeted the short isoform of the zinc‐finger antiviral protein (ZAP‐S) and bound to specific residues (E111, E115, and F549). This result was confirmed using cellular thermal shift assays, bio‐layer interferometry, and mutagenesis studies. This interaction enhanced the ZAP‐S stability and disrupted –1 programmed ribosomal frameshifting (–1PRF), a critical process for viral polyprotein synthesis. The antiviral activity of compound **7** was ZAP‐S‐dependent, as ZAP‐S knockdown abolished its efficacy while overexpression enhanced it. These results established compound **7** as a novel antiviral candidate that can combat SARS‐CoV‐2 and its variants by targeting ZAP to inhibit –1PRF. This compound, therefore, represents a promising therapeutic strategy.

## Graphical abstract

The present study evaluated the antiviral potential of various lycorine derivatives and identified compound **7** as a promising candidate. Compound **7** demonstrated strong antiviral efficacy against both the original SARS‐CoV‐2 and its variants in vitro and in vivo. Mechanistically, compound **7** exerts its antiviral effects by targeting the ZAP protein and disrupting the critical –1PRF process of SARS‐CoV‐2, thereby impeding viral replication. These findings suggest compound **7** as a novel antiviral candidate with significant potential for combating SARS‐CoV‐2 and its emerging variants.

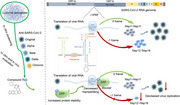

## 1 Introduction

SARS‐CoV‐2 variants are continuously emerging, and this is a formidable and ongoing challenge to global public health. Hence, there is an urgent need to develop specific antiviral drugs against COVID‐19 [1, 2, 3]. The approximately 30 kb positive‐sense RNA genome of SARS‐CoV‐2 encodes accessory proteins, structural proteins, and two polyproteins (PPs) translated from the open reading frames 1a (ORF1a) and ORF1b [4, 5]. ORF1a translation directly produces the pp1a polyprotein. However, through a –1PRF event, ribosomes can slip back one nucleotide on the RNA and continue translation into ORF1b, thereby generating the pp1ab polyprotein. Sixteen nonstructural proteins (Nsp1–16) are generated from the proteolytic processing of pp1a and pp1ab, and these assemble to form the replication–transcription machinery. Specifically, ORF1a encodes pp1a, which is cleaved to produce Nsp1–Nsp11, and ORF1b encodes pp1ab, which is cleaved to produce Nsp12 to Nsp16 [6, 7, 8, 9]. The utilization of –1PRF in SARS‐CoV‐2 is indispensable to ensure the expression of pivotal proteins, such as RNA‐dependent RNA polymerase (RdRp), which is vital for synthesizing viral RNA during propagation [7, 8]. Any –1PRF efficiency alteration would consequently impair viral replication, and this mechanism has emerged as an attractive broad‐spectrum antiviral target [9, 10].

−1PRF efficiency is a crucial step in the replication cycle of SARS‐CoV‐2, and it is modulated by host factors such as ZAP [12] and SHFL [13]. Importantly, the zinc‐finger antiviral protein (ZAP) is an interferon‐induced factor with two isoforms (the long ZAP‐L and the short ZAP‐S). ZAP can directly bind the SARS‐CoV‐2 frameshift‐stimulatory element (FSE). In particular, its short isoform ZAP‐S suppresses −1PRF, thereby restricting viral replication. Based on the strategy of targeting –1PRF as a drug target, some small‐molecule compounds with potential antiviral activity have been successfully screened, including merafloxacin [14] and geneticin [15]. However, pharmacological strategies that enhance intrinsic host restriction factors, such as ZAP, remain comparatively underexplored.

Natural products provide a rich source of antiviral scaffolds [16, 17, 18]. Lycorine is a plant‐derived alkaloid that has shown broad antiviral activities against coronavirus, flavivirus, retrovirus, and enterovirus [19, 20]. Several studies have demonstrated that lycorine exhibits antiviral activity against SARS‐CoV‐2 in vitro [21, 22, 23, 24, 25, 26, 27]. Indeed, it has also been suggested that lycorine may impede –1PRF, thereby affecting SARS‐CoV‐2 replication [26].

In the present study, we designed and synthesized a series of new lycorine derivatives and identified a lead molecule, compound **7**. This compound was found to possess an improved therapeutic window in vitro and definite in vivo antiviral activity. Mechanistic studies showed that compound **7** impaired the −1PRF process by directly binding to ZAP‐S (E111, E115, and F549). This interaction enhanced ZAP‐S stability and thereby inhibited frameshifting efficacy. This capacity highlighted its potential as a drug candidate for the clinical development of new antiviral agents.

## 2 Results

### 2.1 Synthesis and Structural Confirmation of Lycorine Derivatives

**FIGURE 1:**
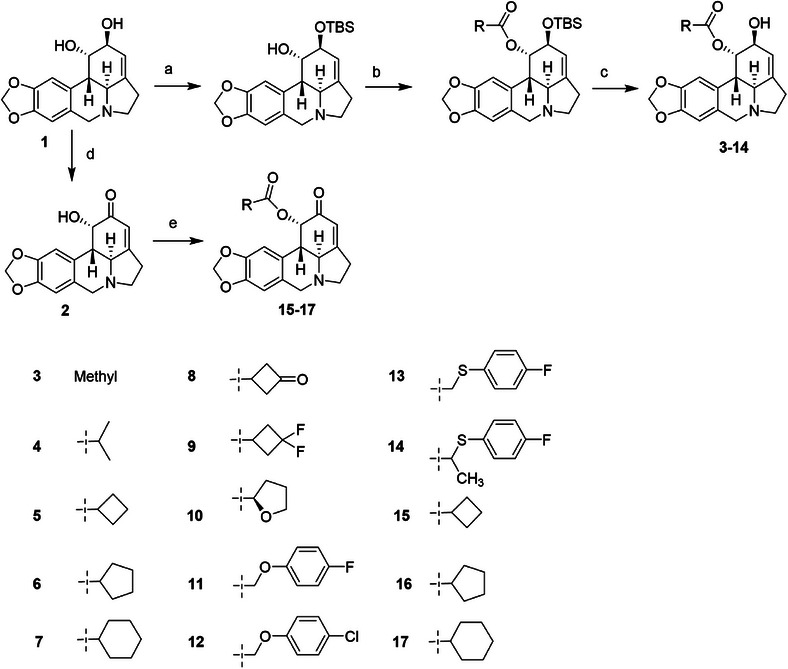
Synthesis and structural confirmation of lycorine derivatives. Synthetic routes to selected lycorine derivatives (**3**–**17**). *Reagents and conditions*: (A) imidazole, TBSCl, DMF, r.t.; (B) EDCI, DMAP, acid, DCM; (C) (1)12N HCl aq, EtOH, r.t.; (2) sat.Na_2_CO_3_ aq; (D) Dess–Martin reagent, pyridine, DMF; (E) Acid, EDCI, DMAP, DCM.

All of the lycorine derivatives were synthesized, as shown in Figure 1. Lycorine was first reacted with *tert*‐butyl dimethyl chlorosilane (TBSCl) in *N*, *N*‐dimethylformamide (DMF) and pyridine to form a 2‐TBS‐protected hydroxyl lycorine derivative. This was then reacted with the corresponding carboxylic acid in 4‐dimethylaminopyridine (DMAP) and 1‐ethyl‐(3‐dimethylaminopropyl) carbodiimide hydrochloride (EDCI) in a dichloromethane solvent to obtain 1‐hydroxyl lycorine ester, which was deprotected to obtain the target derivatives **3–14**. Alternatively, lycorine was first oxidized with Dess–Martin periodinane reagent to obtain compound **2**, which was then reacted with the corresponding carboxylic acid in EDCI and DMAP in a dichloromethane solvent to obtain derivatives **15–17**. The synthesis methods and details of each compound are provided in Figure S1.

### 2.2 Lycorine Derivatives Exhibit Potent Antiviral Activity Against SARS‐CoV‐2 and Its Variants In Vitro

**FIGURE 2:**
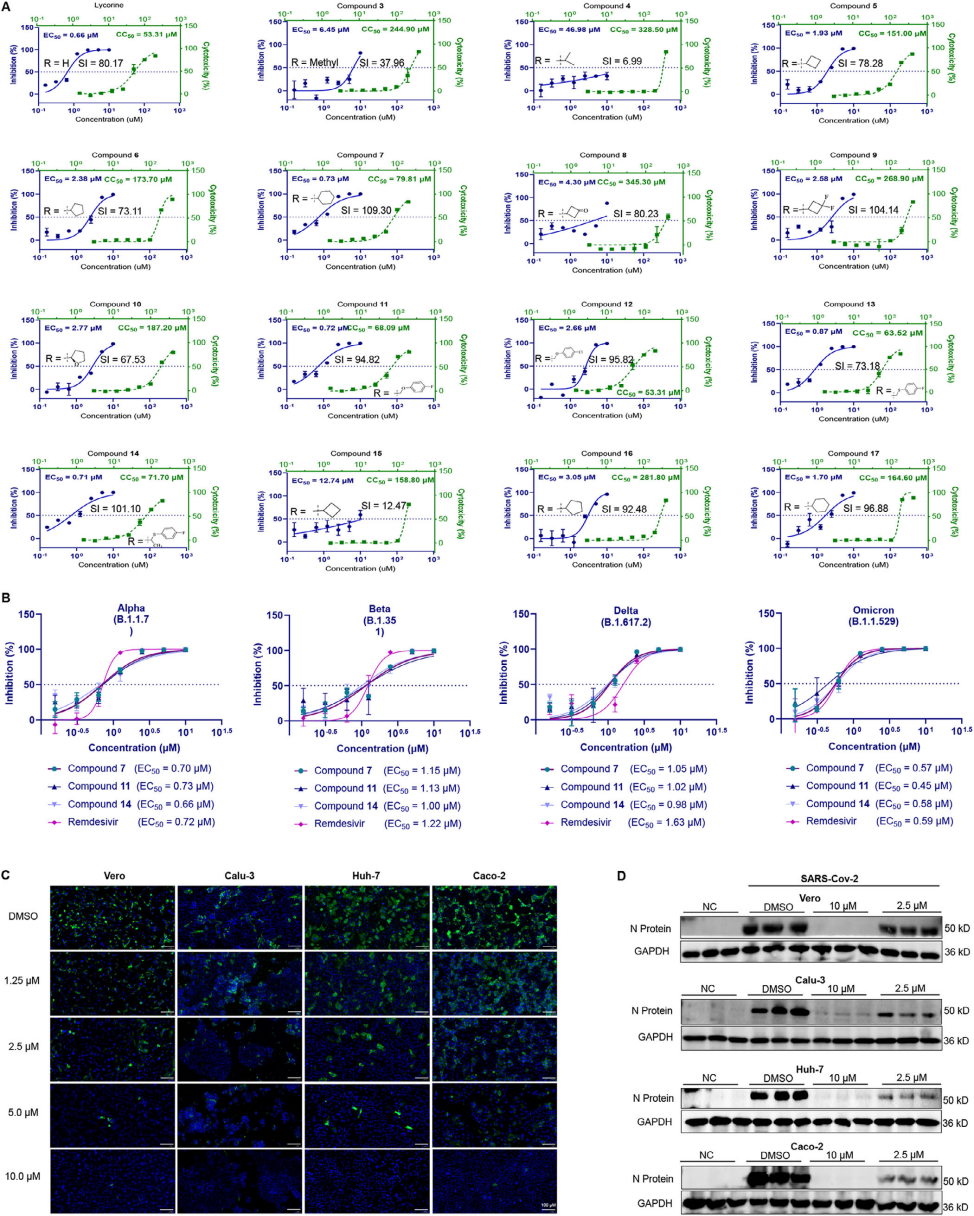
Lycorine derivatives exhibit potent antiviral activity against SARS‐CoV‐2 and its variants in vitro. (A) Dose‐dependent inhibition of SARS‐CoV‐2 original strain (GD108) infection by selected lycorine derivatives. Vero cells infected with SARS‐CoV‐2 at MOI of 0.05 were treated with serially diluted compounds for 48 h. Viral RNA copy numbers in culture media were quantified by RT‐qPCR (real‐time quantitative PCR). The dose‐inhibition curve for each compound is shown above the corresponding plot (*n* = 3). Cytotoxicity assay of lycorine derivatives. Vero cells were treated with lycorine derivatives at gradient concentrations for 48 h. Cytotoxicity was assayed by CCK‐8 (*n* = 3). (B) Vero cells were infected with different strains of SARS‐CoV‐2 (Alpha, Beta, Delta, and Omicron BA.1) at MOI of 0.05 and treated with serially diluted compounds (**7**, **11**, **14**, and remdesivir) for 48 h. Viral RNA copy numbers in culture media were quantified by RT‐qPCR. The EC_50_ value for each compound is shown above the corresponding plot (*n* = 3). (C) IF of the inhibition of compound **7** on SARS‐CoV‐2 replication in Vero, Calu‐3, Huh‐7, and Caco‐2. At 48 h, the infected cells (MOI = 0.05) treated with different concentrations of compound **7** were fixed and analyzed by IF using the primary antibody against SARS‐CoV‐2 N protein (Green). Cell nuclei were stained with DAPI (blue). Scale bars = 100 µm. (D) WB analysis of the inhibition of compound **7** on SARS‐CoV‐2 replication in Vero, Calu‐3, Huh‐7, and Caco‐2. The cells were infected with SARS‐CoV‐2 GD108 at MOI of 0.05 and then treated with compound **7**. N protein expression was detected by WB at 48 h post‐infection (*n* = 3).

The inhibitory effects of the lycorine derivatives were then analyzed against the original SARS‐CoV‐2 strain. The 50% maximal effective concentrations (EC_50_) of most derivatives (compounds **3**, **5–16**) were less than 10 µM. The EC_50_ values of compounds **7**, **11**, **13**, and **14** were less than 1 µM, with values of 0.73, 0.72, 0.87, and 0.71 µM, respectively. These were equivalent to the value of lycorine (0.66 µM) (Figure 2A). The 50% toxicity concentration (CC_50_) curves are shown in Figure 2A. The CC_50_ values of lycorine and compounds **7**, **11**, **13**, and **14** were 53.31, 79.81, 68.09, 65.32, and 71.70 µM, respectively. The selectivity index (SI) values of compounds **7**, **11**, and **14** were 109.30, 94.82, and 101.10, respectively, and these were greater than that of lycorine (80.17). These results suggested that these three derivatives would exhibit comparable antiviral activity to lycorine and provide a wider therapeutic window. The inhibitory effect of the lycorine derivatives on SARS‐CoV‐2 variants of concern (VOCs) was investigated by determining the activity of compounds **7**, **11**, and **14** against four VOCs: Alpha, Beta, Delta, and Omicron. Our results showed that compounds **7**, **11**, and **14** exhibited promising antiviral activities against all of the tested SARS‐CoV‐2 VOCs (Figure 2B). Compound **7** emerged as the most promising candidate based on its highest SI value and was selected for further assessment. To assess its safety profile, we compared the cytotoxic effects of lycorine and compound **7** across Huh‐7, H1299, and CP‐H209 cell lines (Table S1). The data clearly showed that compound **7** displayed a reduced cytotoxicity relative to lycorine in all of the tested cell lines. This result supported its enhanced safety properties. To comprehensively examine the antiviral efficacy of compound **7** beyond the Vero cells, we extended our assessment to three additional human cell lines: Calu‐3, Huh‐7, and Caco‐2. The antiviral activity of compound **7** was assessed using immunofluorescence (IF) and western blot (WB) analyses. Notably, compound **7** treatment at either a lower (2.5 µM) or a higher (10 µM) concentration significantly decreased SARS‐CoV‐2 nucleocapsid (N) protein expression in a majority of the tested cell lines. Collectively, these results underscore the broad‐spectrum antiviral activity of compound **7** against SARS‐CoV‐2 across various cell types (Figure 2C,D). Further, the RNA‐seq analysis revealed that compound **7** treatment resulted in a significant reversal of SARS‐CoV‐2‐induced host gene expression changes in infected Calu‐3 and Huh‐7 cells (Figure S2). These results strongly suggested that compound **7** would have a significant efficacy against SARS‐CoV‐2.

### 2.3 Compound 7 Decreases the Viral Load and Pathological Injury in a Hamster Model of Omicron Infection

**FIGURE 3:**
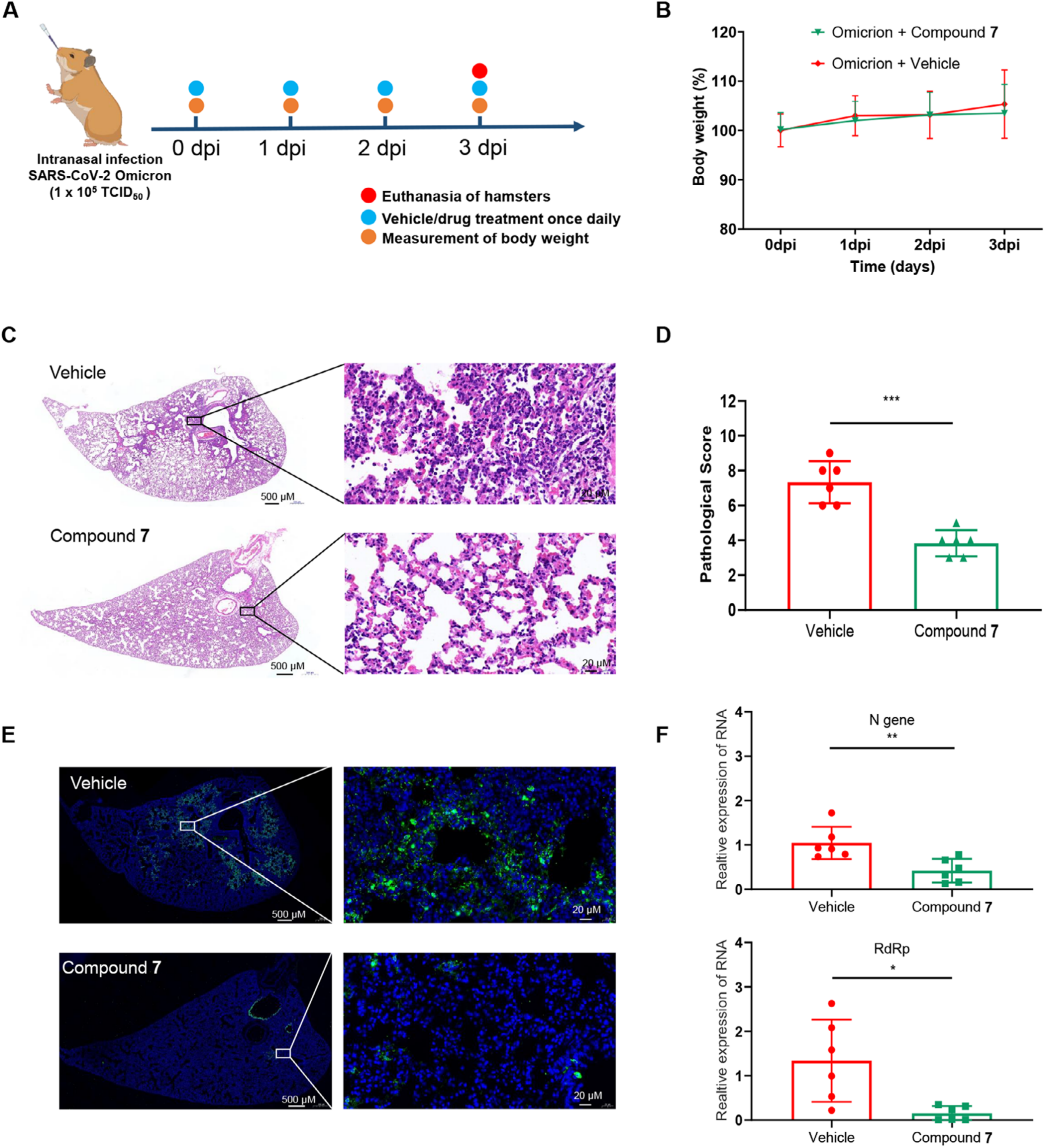
Compound 7 decreases the viral loads and pathological injury in hamster models of Omicron. (A) Schematic diagram of experimental design. Hamsters (*n* = 6 per group) were intranasally infected with 10^5^ TCID_50_ of SARS‐CoV‐2 Omicron BA.2. (B) Body weight changes. Changes in body weight were recorded over the entire study period (*n* = 6). (C) Lung histopathology. Representative H&E‐stained sections of left lung tissues harvested at day 3 postinfection are shown (*n* = 6). (D) Histopathological scores of lung injury of compound **7**‐treated SARS‐CoV‐2‐infected hamsters (*n* = 6). (E) Representative images of IF staining for SARS‐CoV‐2. The fluorescence of N protein (green) in lung tissues of vehicle or compound **7**‐treated COVID‐19 hamster (*n* = 6). (F) Viral RNA levels in the right lung tissues collected at 3 dpi were measured by RT‐qPCR using primers targeting the SARS‐CoV‐2 N and RdRp (*n* = 6).

Syrian hamsters were randomly divided into vehicle and compound **7** treatment groups and intranasally challenged with Omicron BA.2 at a median tissue culture infectious dose (TCID_50_) of 10^5^ to test the antiviral effect of compound **7** against SARS‐CoV‐2 in vivo. Syrian hamsters were administered the compound **7** (10 mg/kg) treatment intranasally once daily after the intranasal challenge with SARS‐CoV‐2 (Figure 3A). The hamsters showed no clinical symptoms, and their body weight did not significantly change during the study period (Figure 3B). After treatment with compound **7**, lung lesions were reduced compared with those in the vehicle group, and the widening of the alveolar septum, bronchi, and perivascular interstitium, as well as lymphocyte and neutrophil infiltration, were decreased (Figure 3C; Figure S3). Lung histopathology was assessed by grading the injuries (Figure 3D). The SARS‐CoV‐2 N protein expressions in the hamster lungs were then measured using IF. The N protein signal in the compound **7** group was significantly lower than that in the vehicle group, as shown in Figure 3E and Figure S3. Further, the viral RNA levels in the right lung tissues were quantified using RT‐qPCR that targeted the *N* and *RdRp* genes using *Actin* mRNA as the internal control. The results showed that compound **7** reduced lung viral RNA levels (Figure 3F). Collectively, these results demonstrated that compound **7** inhibited SARS‐CoV‐2 replication in infected hamster lungs.

### 2.4 Compound 7 Reduces −1PRF by Targeting ZAP

**FIGURE 4:**
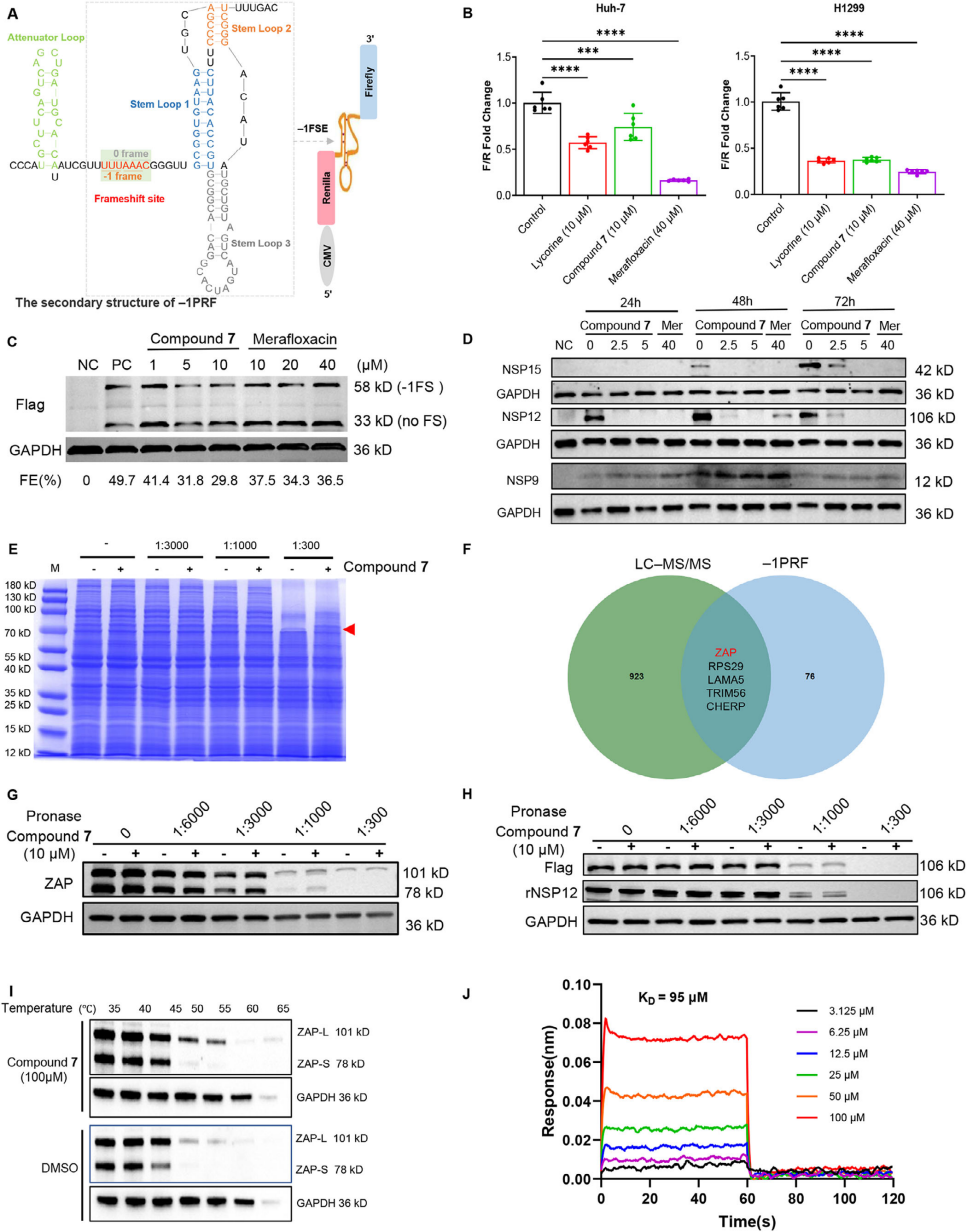
Compound 7 directly binds to ZAP and leads to a decrease in –1PRF. (A) Schematic representation of the dual‐luciferase frameshift reporter construct. The coding sequences for *Renilla* luciferase and *Firefly* luciferase were separated by the SARS‐CoV‐2 –1FSE sequence (13460‐13548). (B) Fold change in the Firefly/Renilla (F/R) luciferase ratio after treatment with the indicated drugs (lycorine at 10 µM, compound **7** at 10 µM, merafloxacin at 40 µM). Huh‐7 and H1299 cells transfected with the pHRF‐FSE (–1) luciferase reporter vector were treated with DMSO (Control) or the indicated drugs for 48 h (*n* = 6 per group). (C) The frameshift Reporter mRNA containing a 3×FLAG‐tag followed by nucleotides 12686–14190 of the SARS‐CoV‐2 genome was translated in a RRL translation system in the presence of compound **7** or merafloxacin. The 3×FLAG‐tag was introduced at the N‐terminus to facilitate detection. WB analysis of the compound **7** effect on the –1PRF frameshift efficiency (FE) using anti‐flag antibody (Sigma, F3165). BC (blank control), NC (negative control). (D) Relative abundance of Nsp9, Nsp12, and Nsp15 in compound **7** (0, 2.5, and 5 µM) or Mer (merafloxacin, 40 µM)‐treated Vero cells after SARS‐CoV‐2 infection (MOI = 0.05). (E) The cellular target of compound **7** was identified using DARTS technology coupled with LC–MS/MS in H1299 cells. M, marker. (F) Venn diagram between the target proteins of compound **7** and the in vitro RNA antisense purification of the SARS‐CoV‐2 frameshift site from the literature. (G) ZAP protein stability was increased upon compound **7** (10 µM) treatment in H1299 cell lysates. (H) H1299 cells were transfected with the pcDNA3.1‐3×Flag‐Nsp12 plasmid and cultured for 48 h. Cells were then lysed and treated with 10 µM compound **7** (+) or DMSO (–). Recombinant Nsp12 (rNsp12) protein was detected by WB analysis, which was performed using anti‐Flag and anti‐Nsp12 antibodies for detection. (I) CETSA confirmed the binding of compound **7** (50 µM) to ZAP in 293T cells, with GAPDH serving as the internal control. (J) The binding of compound **7** to ZAP was depicted through BLI.

It has been suggested that lycorine inhibits –1PRF and consequently affects SARS‐CoV‐2 replication [26]. The FSE is a highly conserved mRNA region in −1PRF that is required for the translation of SARS‐CoV‐2 PPs [28, 29]. To investigate the effect of compound **7** on SARS‐CoV‐2 frameshifting, the pHRF‐FSE (–1) vector was used as a dual luciferase reporter to detect the –1PRF efficiency (Figure 4A). Our results showed that compound **7** inhibited SARS‐CoV‐2 frameshifts in Huh‐7 and H1299 cells (Figure 4B). In addition, we also constructed dual‐luciferase reporter vectors containing the –1PRF sequences specific to MERS‐CoV and SARS‐CoV‐1. Our experimental results revealed that compound **7** significantly suppressed the –1PRF efficiency in both MERS‐CoV and SARS‐CoV‐1. This was similar to its observed effects on SARS‐CoV‐2. This suggested that compound **7** may possess broad‐spectrum antiviral activity by interfering with a mechanism that is essential for viral replication and conserved across these coronaviruses (Figure S4). We then examined the effect of compound **7** on –1PRF efficiency by analyzing the protein products obtained through an in vitro translation assay using the rabbit reticulocyte lysate (RRL) translation system in the presence or absence of compound **7**. The detection of translation products was performed using WB with an anti‐flag antibody. The frameshifting efficiency (FE) was determined as the ratio of the frameshift product relative to the total of both the frameshift (FS) and no frameshift (no FS) products. Compound **7** inhibited SARS‐CoV‐2 frameshifting in a dose‐dependent manner. The compound **7** treatment reduced the FE to 41.4% at 1 µM, 31.8% at 5 µM, and 29.8% at 10 µM (Figure 4C) relative to the negative control (NC, FE = 49.7%). These results demonstrated that compound **7** effectively disrupted the –1PRF‐mediated switch from ORF1a to ORF1b translation. We then assessed the inhibitory effect of compound **7** on viral protein synthesis encoded by the ORF1a and ORF1b sequences in SARS‐CoV‐2‐infected Vero cells. Following viral infection and subsequent compound **7** treatment, as shown in Figure 4D, the Nsp12 and Nsp15 (normalized to Nsp9) relative levels were significantly reduced at different time points (12, 24, and 48 h) postinfection. These results suggested that compound **7** exerted inhibitory effects on SARS‐CoV‐2 replication by attenuating –1PRF efficiency.

Identification and exploration of protein–ligand interactions can reveal the crucial roles regarding mechanisms of drug action and the biology of disease‐related targets. To identify the direct proteins that mediate the antiviral properties of compound **7**, drug affinity responsive target stability (DARTS) was used to identify its direct targets by analyzing the differential proteins after exposure to compound **7**. The results showed a strongly protected band at approximately 80 kDa following treatment with 1:300‐diluted pronase, as shown in Figure 4E. The 1:300 proteolyzed samples treated with or without 100 µM compound **7** were then analyzed and identified using mass spectrometry (MS). The MS data showed that 927 proteins exhibited an enrichment level that was at least twice that of the untreated control group (Table S2). Notably, five of these proteins had also been implicated in SARS‐CoV‐2 frameshift site RNA interactions, as reported in a recent study [12] (Figure 4F; Table S3). Among these potential targets, the ZAP protein has emerged as a focal point due to its unique identity as a zinc finger antiviral protein that plays a vital role in antiviral defense [30, 31, 32, 33, 34]. Studies have demonstrated that ZAP inhibits SARS‐CoV‐2 replication through its high binding affinity with SARS‐CoV‐2 RNA [35, 36, 37]. Therefore, the ZAP protein was selected as a potential target of compound **7** for further validation. We found that compound **7** treatment in H1299 cell lysates significantly enhanced ZAP stability, particularly for its short isoform (Figure 4G). ZAP is a protein with two isoforms, ZAP‐L and ZAP‐S (Figure 4H). The short ZAP‐S isoform has been shown to play a pivotal role in antiviral activity by inhibiting the –1PRF of SARS‐CoV‐2 [12]. Therefore, the ZAP‐S and compound **7** interaction was prioritized in this study. A cellular thermal shift assay (CETSA) was conducted in 293T cells to verify the compound **7** and ZAP‐S interaction and confirm our observations. The ZAP‐S thermal stability was enhanced to different degrees with the addition of compound **7** (50 µM) across a temperature range of 35°C to 65°C (Figure 4I). This result indicated a possible direct interaction. To rigorously establish the specificity of this interaction, we used bio‐layer interferometry (BLI) to examine the binding affinity between the purified ZAP‐S protein and compound **7**. The results definitively showed that compound **7** bound to ZAP‐S with a moderate affinity. This was characterized by a dissociation constant (KD) of 95 µM (Figure 4J). This result confirmed that ZAP‐S was indeed a direct target of compound **7**. We then hypothesized that compound **7** impeded viral replication by targeting ZAP‐S to suppress the –1PRF process of SARS‐CoV‐2.

### 2.5 E111, E115, and F549 Are Key Binding Sites of ZAP‐S to Compound 7

**FIGURE 5:**
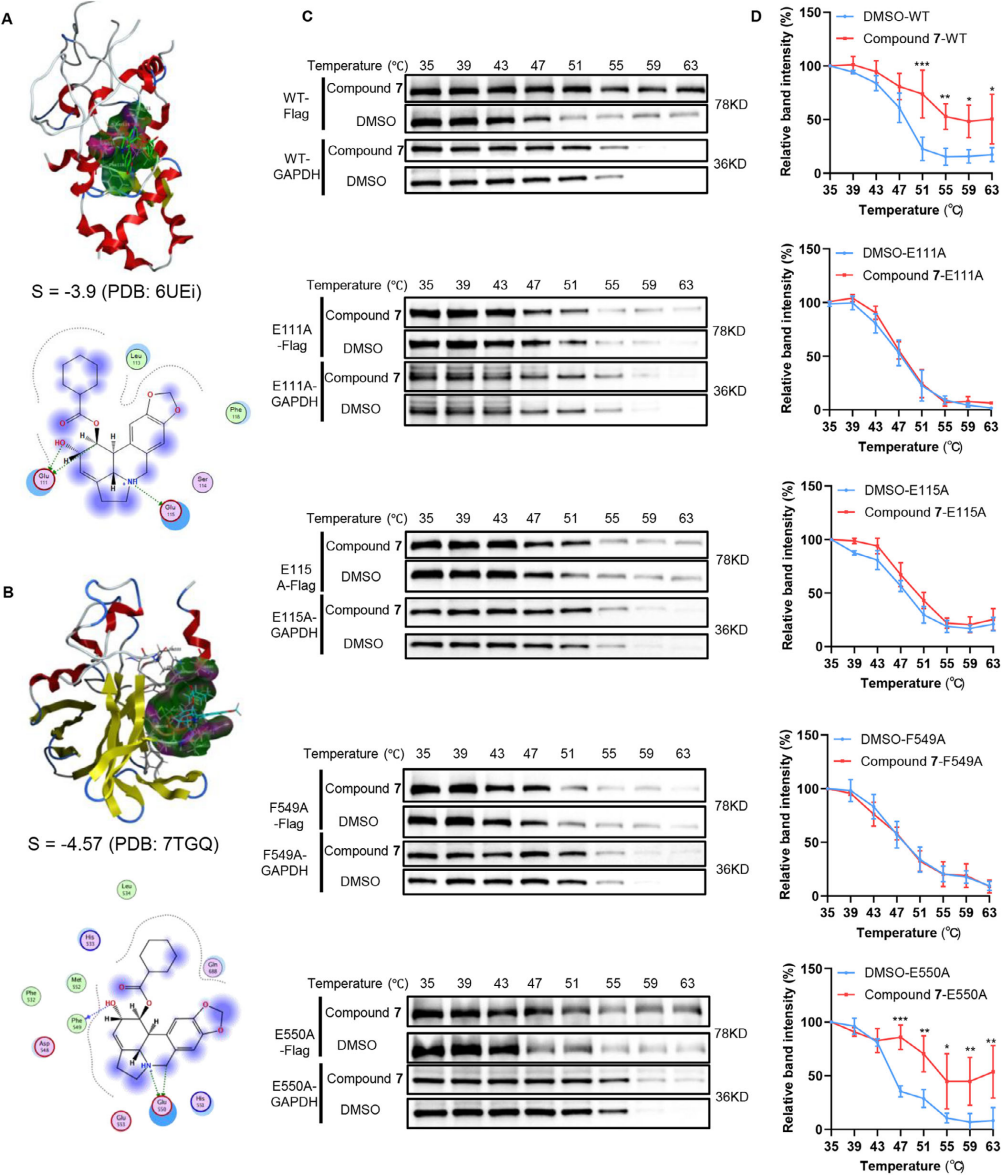
E111, E115, and F549 are key binding sites of ZAP‐S for compound 7. (A) Molecular docking results for N‐terminal residues of ZAP (PDB code: 6UEI) with compound **7** (top). The 2D image reveals that compound **7** interacts with amino acids Glu111 and Glu115 (bottom). (B) Molecular docking results for the central domain of ZAP (PDB code: 7TGQ) with compound **7** (top). The 2D image reveals that compound **7** interacts with several amino acids, including Phe549 and Glu550 (bottom). (C) CETSA analysis of the thermal stability of ZAP‐S^WT^, ZAP‐S^E111A^, ZAP‐S^E115A^, ZAP‐S^F549A^, and ZAP‐S^E550A^ after treatment with compound **7** (50 µM). (D) The CETSA curve and the thermal stability were performed using GraphPad Prism software. *n* = 3 per group.

To identify the specific binding sites of compound **7** on ZAP, four amino acid residues, namely, E111, E115, F549, and E550, which were predicted using docking simulations, were selected as critical interaction points (Figure 5A,B; Table S4). The molecular docking results for ZAP with lycorine are shown in Figure S5. To experimentally validate the significance of these residues for compound **7** binding, we constructed a series of mutant ZAP‐S‐expressing plasmids by substituting the predicted amino acids with alanine: pcDNA4.0‐3*flag‐ZAP‐S^E111A^(Mut1), pcDNA4.0‐3*flag‐ZAP‐S^E115A^(Mut2), pcDNA4.0‐3*flag‐ZAP‐S^F549A^(Mut3), and pcDNA4.0‐3*flag‐ZAP‐S^E550A^(Mut4). These mutants and the wild‐type (WT) ZAP‐S‐expressing plasmid were then transfected into 293T cells to examine their effect on the interaction with compound **7**. CETSAs and WBs were then conducted to assess the thermal stability of the WT and mutant ZAP‐S proteins in the presence and absence of compound **7**. Our results revealed that while WT ZAP‐S exhibited increased thermal stability upon compound **7** binding, this stabilization was absent in Mut1, Mut2, and Mut3 (Figure 5C,D). This observation underscored the critical role of the E111, E115, and F549 residues for mediating the direct binding of compound **7** to ZAP‐S.

### 2.6 Compound 7 Exerts Antiviral Efficacy Dependent Upon ZAP‐S

**FIGURE 6:**
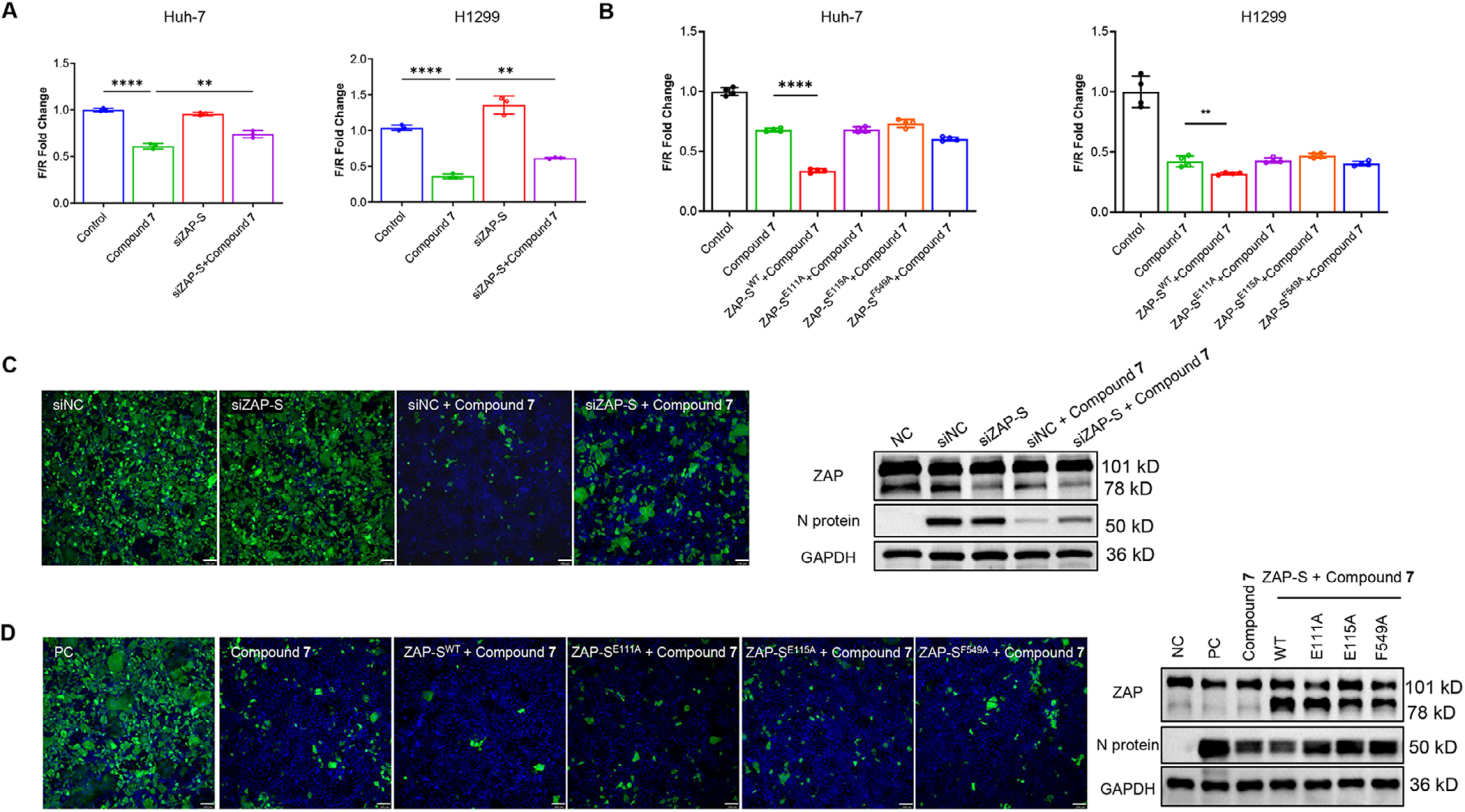
Compound 7 exerts antiviral efficacy dependent upon ZAP‐S. (A) Effects of ZAP‐S knockdown on –1PRF followed by DMSO or compound **7** (10 µM) treatment in Huh‐7 and H1299 cells (*n* = 3). (B) Compound **7** (10 µM) in combination with ZAP overexpression synergistically decreased –1PRF in H1299 and Huh‐7 cells (*n* = 4); (C and D) Antiviral activity of compound **7** (2.5 µM) following ZAP‐S knockdown, or overexpression of ZAP‐S and mutant ZAP‐S. IF visualization of SARS‐CoV‐2 N protein (green) and cell nuclei (blue) in infected Huh‐7 cells at 48 h posttreatment (left). Scale bar: 100 µm. WB of N protein expression in Huh‐7 cells infected with SARS‐CoV‐2 (right).

We wanted to further validate the hypothesis that the antiviral mechanism of compound **7** relies on its binding to ZAP‐S. Therefore, we then conducted a comprehensive rescue experiment. We first manipulated the ZAP‐S expression in Huh‐7 and H1299 cells by transfecting siRNA that targets ZAP‐S to knock down its endogenous expression, a flag‐tagged WT ZAP‐S plasmid, or binding‐defective mutant ZAP‐S plasmids. After the performance of these manipulations, the cells were transfected with a dual luciferase reporter system (pHRF‐FSE (–1)) and exposed to compound **7**. Our observations revealed that ZAP‐S knockdown diminished the inhibitory potential of compound **7** on –1PRF, whereas ZAP‐S overexpression bolstered its effectiveness. We then asked whether ZAP‐S would be required for the antiviral efficacy of compound **7** (Figure 6A). In contrast, the ZAP‐S mutant variants did not show this effect (Figure 6B). We then extended our investigation to the antiviral activity of compound **7** against SARS‐CoV‐2. Huh‐7 cells were transfected with siRNA‐ZAP, wild‐type (WT) ZAP‐S, or mutant ZAP‐S plasmids. After SARS‐CoV‐2 infection, cells were treated with 2.5 µM of compound **7** for 48 h, and the viral replication was analyzed using IF and WB. The results indicated that the ZAP‐S knockdown significantly impaired the antiviral activity of compound **7**. This was evidenced by the increased viral loads compared with the control. Conversely, WT ZAP‐S overexpression fortified the antiviral response to compound **7**, whereas the ZAP‐S mutant variants failed to do this (Figure 6C,D). These comprehensive findings strongly suggested that compound **7** exerted its antiviral activity through direct interaction with ZAP‐S, thereby inhibiting SARS‐CoV‐2 –1PRF and effectively restricting viral replication.

## 3 Discussion

Lycorine is a potent antiviral agent that exhibits inhibitory effects against a wide range of viruses, including arboviruses (ZIKV, DENV, and CHIKV) [38, 39, 40, 41, 42], enteroviruses (EV71 and CA16) [43, 44, 45], and coronaviruses (MERS‐CoV, SARS‐CoV, and SARS‐CoV‐2) [21, 22, 23, 24, 25, 26, 27, 46, 47, 48, 49, 50, 51]. In response to the metabolic instability of lycorine in vivo, different types of structural derivatives of lycorine were designed and synthesized in this study (Figure S1). The preliminary screening of antiviral activity showed that only lycorine and its esters, as well as 2‐carbonyl lycorine and its esters, showed antiviral activities against SARS‐CoV‐2. All of the other structural compounds that were tested were inactive. Previous studies have shown that the 1‐hydroxyl esterification of lycorine can delay the lycorine release rate in the body, prolong its half‐life, and mitigate its toxic side effects [52, 53]. Seven lycorine derivatives (compounds **7**, **9**, **11**, **12**, **14**, **16**, and **17**) exhibited greater SI values than lycorine according to the primary screening against SARS‐CoV‐2. The preliminary structure–activity relationship showed that the cyclic carboxylic acid ester of 1‐hydroxylycorine (**5**, **6**, **7**) had better activity than the chain carboxylic acid (**3**, **4**). In cyclic carboxylic acid esters, the SI value was greater for the hexagonal than for the pentagonal and quaternary rings (**5**, **6**, **7**). 4'‐Fluorophenoxyacetoacetate 1‐hydroxylycorine (**11**) showed better activity than 4'‐chlorophenoxyacetate (**12**), and 4'‐fluorophenoxypropionate 1‐hydroxylycorine (**14**) showed better activity than 4'‐fluorophenoxyacetate (**13**). 2‐carbonylycorine esters derivatives with cyclic carboxylic acids also exhibited good structure–activity relationships, with EC_50_ and SI values decreasing from those with six‐membered rings to five‐membered rings and to four‐membered rings (**15**, **16**, **17**). We identified several lycorine derivatives with promising antiviral activity. Compounds **7**, **11**, and **14** exhibited strong inhibitory effects against both SARS‐CoV‐2 and its variants, including Alpha, Beta, Delta, and Omicron. Compound **7** stood out due to its high SI, indicating potent antiviral activity. Further, compound **7** exhibited significant antiviral activity against SARS‐CoV‐2 in both in vitro and in vivo experiments.

We investigated the potential effect of compound **7** on –1PRF, a critical process for SARS‐CoV‐2 replication [6, 7, 8, 9], to elucidate the mechanism underlying its antiviral activity. We confirmed that compound **7** markedly reduced the frameshift efficiency of –1PRF. Further research demonstrated that the inhibitory effect of compound **7** on –1PRF depended on the ZAP‐S protein. The ZAP protein, also known as ZC3HAV1, plays a critical role as a host antiviral factor. It is essential for the defense against various viral infections [34, 54, 55, 56, 57]. Its unique CCCH‐type zinc finger structure allows it to recognize and bind to specific RNA sequences, and this is the foundation of its viral replication inhibition [30, 58]. Recent SARS‐CoV‐2 research revealed the role of ZAP for combating this virus [12, 35, 36, 37, 59, 60, 61, 62, 63]. In particular, the ZAP short isoform plays a crucial role in preventing –1PRF of SARS‐CoV‐2 [12]. We demonstrated that compound **7** enhanced ZAP‐S stability and physically interacted with it, as evidenced by the CETSA and BLI results. Additionally, docking simulations and mutagenesis experiments identified E111, E115, and F549 as key binding sites for compound **7** on ZAP‐S. This result further confirmed the specificity of this interaction. Importantly, the manipulation of ZAP‐S expression levels in cells affected the antiviral activity of compound **7**. ZAP‐S expression silencing diminished the inhibitory effect of compound **7** on –1PRF and viral replication. In contrast, ZAP‐S overexpression enhanced its antiviral efficacy. These results strongly suggested that the antiviral activity of compound **7** against –1PRF relied on its interaction with ZAP‐S.

Despite these promising findings, the specific mechanism of action of compound **7** in viral infection remains not yet fully elucidated. First, the ZAP protein exists in two isoforms: ZAP‐L and ZAP‐S. We primarily explored the regulatory mechanism of compound **7** on –1PRF through ZAP‐S, while the role of ZAP‐L remains unexplored. Our results indicated that compound **7** also has a certain impact on ZAP‐L stability, although this effect is slightly weaker than its effect on ZAP‐S (Figure 4G,I). This result suggests that compound **7** partially exerts its antiviral efficacy through its action on ZAP‐L. Therefore, it is necessary to further investigate the specific mechanisms responsible for the antiviral activity of compound **7** via ZAP‐L in future investigations. Second, beyond the ZAP protein, we also identified four additional proteins (CHERP, FAM50A, TRIM56, and RPS29) that may interact with compound **7** and potentially play a role in –1PRF regulation (Figure 4F). An exploration of the antiviral relationships between these proteins and compound **7** would have significant implications for future research. Third, the RNA‐seq data showed that the infected cells treated with compound **7** exhibited a notable reversal of host gene expression alterations induced by SARS‐CoV‐2. Specifically, the NF‐κB and TNF signaling pathways were markedly upregulated following viral infection in Calu‐3 and Huh‐7 cells. These alterations were reversed upon compound **7** treatment (Figure S2). An investigation regarding whether compound **7** exerts its antiviral effect directly through the NF‐κB and TNF signaling pathways or indirectly by influencing these pathways through other mechanisms is a topic that is worthy of further exploration. Finally, compound **7** may involve additional antiviral pathways that require further exploration. To fully comprehend the antiviral activity of compound **7** and its potential clinical applications, more comprehensive and in‐depth research is required.

In summary, a series of lycorine derivatives was evaluated in this study. Compound **7** was determined to be the most promising candidate due to its favorable safety profile and potent antiviral activity against SARS‐CoV‐2. Compound **7** exhibited robust antiviral efficacy against both the original SARS‐CoV‐2 strain and its variants in vitro and in vivo. The mechanistic studies revealed that compound **7** exerted its antiviral effects by targeting the ZAP protein and disrupting the critical –1PRF process of SARS‐CoV‐2, thereby effectively restricting viral replication. These results indicated that compound **7** may be a novel antiviral candidate with significant therapeutic potential against SARS‐CoV‐2 and its emerging variants.

## 4 Materials and Methods

### 4.1 Compounds

Lycorine derivatives were synthesized according to the method [64] used at the Medicinal Chemistry Laboratory of the Institute of Materia Medica, Chinese Academy of Medical Sciences. We achieved a purity that exceeded 98.0%. These compounds were then diluted to their final working concentrations as described in the experiments.

### 4.2 Cell Lines

Vero cells are an established monkey kidney epithelial cell line. These were obtained from the ATCC (CCL‐81). The human lung adenocarcinoma Calu‐3 cell line (Procell, CL‐0054, Wuhan, China), human hepatoma Huh‐7 cell line (Procell, CL‐0120), human colon Caco‐2 cell line (Procell, CL‐0050), human lung adenocarcinoma H1299 cell line (Procell, CL‐0165), and human embryonic kidney HEK293T cell line (Procell, CL‐0005) were cultured in standard medium at 37°C and 5% CO_2_.

### 4.3 Viruses

Five strains of SARS‐CoV‐2 were selected, including the original strain (GD108) and four VOCs. Specifically, the original strain (National Center for Biotechnology Information [NCBI] Reference Sequence: NC_045512.2) and the Beta variant (B.1.351) were sourced from Guangdong Center for Disease Control (CDC), the Alpha variant (B.1.1.7) from China CDC, the Delta variant (B.1.617.2) from Chongqing CDC, the Omicron BA.1 variant (B.1.1.529) from the Institute of Laboratory Animal Sciences, Chinese Academy of Medical Sciences, and the Omicron BA.2 variant from the Institute of Medical Biology, Chinese Academy of Medical Sciences.

### 4.4 Syrian Hamsters

Adult male Syrian hamsters, aged 6–8 weeks and sourced from Vital River Animal Technology Co. Ltd. in Beijing, were housed two per cage under controlled conditions: a 12/12 h light/dark cycle maintained at a constant temperature of 23°C ± 2°C, with unlimited access to food and water. All of the experiments that involved these animals adhered to the established standards for the ethical use and care of laboratory animals.

### 4.5 Cytotoxicity Assay

To investigate the cytotoxicity of these compounds, Vero cells were inoculated into 96‐well plates at a density of 20,000 cells per well and treated with a gradient concentration of the drug for 48 h. The cytotoxicity was evaluated using a cell counting kit‐8 (CCK‐8) assay (DOJINDO, CK04, Japan). The CC_50_ values of the compounds were calculated using GraphPad Prism software.

### 4.6 Antiviral Activities of Lycorine Derivatives in Vitro

Vero cells were pre‐inoculated on 96‐well plates (20,000 cells/well) and treated with a medium that contained a gradient concentration of compound (10, 5, 2.5, 1.25, 0.625, 0.3125, and 0.15625 µM) in 100 µL/well for 1 h the next day. The cells were then inoculated with SARS‐CoV‐2 for 1 h at a multiplicity of infection (MOI) of 0.05. The supernatant was then removed, and the fresh medium that contained a gradient concentration of compound (10, 5, 2.5, 1.25, 0.625, 0.3125, and 0.15625 µM) in 200 µL/well was added. At 48 h, the antiviral activities were evaluated by measuring the number of viral copies in the cellular supernatant using RT‐qPCR. The drug inhibition rates were then determined based on these viral copy numbers, with the EC_50_ values calculated using GraphPad Prism software.

### 4.7 Syrian Hamster Infection Experiments

Syrian hamsters (6–8 weeks of age) were randomly divided into vehicle or compound **7** treatment groups, and each hamster was intranasally infected with 1 × 10^5^ TCID_50_ SARS‐CoV‐2 Omicron BA.2 strain at 0 dpi. Following infection, the model group received only the vehicle (hydroxypropyl cyclodextrin aqueous solution), whereas the remaining groups received the clathrate compound of **7** and hydroxypropyl cyclodextrin (1:6) (clathrate compound aqueous solution). The Syrian hamsters were administered compound **7** intranasally (10 mg/kg body weight per hamster) once daily for three days after the intranasal challenge with Omicron. All of the hamsters were euthanized on day 3 postinfection, and the body weight loss, lung tissue pathological changes, and the SARS‐CoV‐2 RNA and protein levels in the lungs were measured.

### 4.8 IF Assay

Refer to our previous research for the IF assay method [65]. The primary antibody used in this study was an anti‐SARS‐CoV‐2 N protein, mouse MAb (SinoBiological, 40143‐MM08, mouse, dilution 1:300).

### 4.9 Immunohistopathology

Standard protocols were followed for hematoxylin and eosin (H&E) staining. Images of the stained samples were taken using a panoramic MIDI digital scanner (3DHISTECH, Hungary).

### 4.10 Real‐Time Quantitative PCR

The total RNA was extracted from hamster lung tissues using the TRIzol (TRI) reagent (T9424, Sigma‐Aldrich, USA) according to the manufacturer's instructions. The RNA concentration and quality were determined by measuring the absorbances at 260 and 280 nm using a spectrophotometer (NanoDrop 2000, ThermoFisher Scientific, USA). Values between 1.8 and 2.0 were indicative of high‐quality RNA. The total RNA was obtained using the TaqMan Fast Virus 1‐Step Master Mix to obtain the cDNA. For SARS‐CoV‐2 N gene detection, RT‐qPCR was performed using TagMan Universal PCR Master Mix in a CFX384 Touch Real‐Time PCR detection system (Bio‐Rad, USA) according to the manufacturer's instructions. The primer and probe sequences were the following: forward, GACCCCAAAATCAGCGAAAT; reverse, TCTGGTTACTGCCAGTTGAATCTG; probe, 5′‐FAM‐ACCCCGCATTACGTTTGGTGGACC‐BHQ1‐3′. The viral RNA copies were calculated for EC_50_ using a standard curve produced by continuous 10‐fold dilution of a plasmid that carried the SARS‐CoV‐2 *N* gene. The relative SARS‐CoV‐2 RNA in tissue was determined using *N* and *RdRp* genes, with actin mRNA serving as the internal reference. The primers used are listed in Table S5.

### 4.11 Western Blotting

Standard protocols were followed for western blotting. The primary antibodies used in this study were the anti‐SARS‐CoV‐2 N protein (SinoBiological, 40143‐MM08, mouse, dilution 1:1000), anti‐Nsp9 (GeneTex, GTX636839, rabbit, dilution 1:1000), anti‐Nsp12 (GeneTex, GTX636916, rabbit, dilution 1:1000), anti‐NSP15 (GeneTex, GTX 135738, rabbit, dilution 1:1000), anti‐ZAP (Abcam, ab154680, rabbit, dilution 1:5000), anti‐Flag (Sigma, F3165, mouse, dilution 1:2500), anti‐GAPDH (Proteintech, 10494‐1‐AP, rabbit, dilution 1:5000), and anti‐rabbit or anti‐mouse secondary antibodies (1:10,000, 926–32211 or 926–32210, LI‐COR, USA).

### 4.12 Molecular Docking

The receptors were sourced from the Protein Data Bank (https://www.rcsb.org). The molecular docking model of compound **7** with the co‐crystal structure of the ZAP homology model was performed using Autodock 4.0 software. For the molecular docking studies, these structures underwent independent pre‐processing, which involved the removal of water molecules and the addition of hydrogen atoms. The binding poses were ranked using the docking score.

### 4.13 Dual‐Luciferase Reporter Assay

The dual fluorescence reporter plasmid, pHRF‐FSE (–1), was constructed in our laboratory. In brief, the frameshift sequence of SARS‐CoV‐2 (viral bases 13430–13548), SARS‐CoV‐1 (viral bases 13384–13497), and MERS‐CoV (viral bases13433‐13538) were inserted between the coding sequences of *Renilla* and *Firefly* luciferases. The cells were transfected with the plasmid for 48 h, and the culture medium was supplemented with either a drug or its vehicle. The cells were then lysed, and the lysate was collected for a dual fluorescence assay (Beyotime, RG027, Shanghai, China). The frameshift efficiency was calculated according to the following formula: *Firefly* luciferase luminescence/*Renilla* luciferase luminescence (F/R) = (*Firefly* test/*Renilla* test)/(*Firefly* control/*Renilla* control).

### 4.14 RRL Assay

This assay was conducted using the TNT T7 Coupled Reticulocyte Lysate Systems kit (Promega, L4610). In brief, the pcDNA4.0 SARS‐CoV‐2 reporter plasmid was constructed by inserting the frameshift sequence of SARS‐CoV‐2 (12686–14190) into pcDNA4.0 with an N‐terminal 3×FLAG‐tagged. The plasmid was digested using the restriction endonuclease, XhoI, and purified using a Gel DNA Extraction kit (Vazyme, DC301). The purified linear DNA was used to translate the proteins in vitro. Typical reactions consisted of 50% v/v RRL, 20 µM amino acids, and 0.50 µg template DNA. The tubes were incubated for 1.5 h at 30°C with or without compound **7**. The products were detected using a WB with an anti‐Flag antibody. The –1 and 0 flame blots were detected at 58 and 33 kDa, respectively, and their grayscale was quantified using ImageJ software. The FE was calculated using the following formula: intensity (–1‐frame)/(intensity (–1‐frame) + intensity (0‐frame)).

### 4.15 DARTS Assay

H1299 cells were lysed using a protein extraction reagent (Thermo, 78501), and the cell lysates were incubated with compound **7** or DMSO at room temperature for 1.5 h. The lysates were equally divided, and 1.25 mg/mL of pronase solution (Sigma–Aldrich, 10165921001) served as a stock solution (1:100) and was diluted to 1:300, 1:1000, 1:3000, and 1:6000. The solutions were then added to the lysates at a ratio of 1:25. This was followed by incubation for 30 min. The samples were loaded on SDS/PAGE, and the gel was stained with a PAGE Gel Coomassie Brilliant blue protein staining kit (Solarbio Life Sciences, G4540). The liquid chromatography–tandem mass spectrometry (LC‐MS/MS) analysis was conducted by Shanghai Applied Protein Technology Co., Ltd., China, and the results were analyzed using MaxQuant software (version 1.6.14) with the UniProtKB/SwissProt human database.

### 4.16 Cellular Thermal Shift Assay

After compound **7** treatment or DMSO treatment for 3 h, the HEK‐293T cells were subjected to three freeze–thaw cycles to obtain the cytoplasmic proteins. After centrifugation at 20,000*g* for 30 min at 4°C, the suspension was incubated at 35°C–65°C for 3 min. Following centrifugation at 20,000 g for 30 min at 4°C, the degradation of the indicated proteins at various temperatures was detected using a WB, and the degradation curves of the control and treatment groups were obtained for comparison.

### 4.17 ZAP‐S Protein Purification

The sequence of ZAP‐S (NM_024625.4) with an N‐terminal 6×His tag was optimized, synthesized, and inserted into the pET‐28a vector. The vector was transformed using *E. coli* BL21. This was followed by induction with 0.2 mM isopropyl β‐d‐1‐thiogalactopyranoside at 18°C for 18 h. Prokaryotic‐expressed proteins were captured using Ni‐NTA resin (ThermoFisher Scientific, 88221), eluted with a gradient concentration of imidazole, and enriched by ultrafiltration (Millipore, MWCO 50 kD). The protein was purified using size exclusion chromatography and preserved at –80°C.

### 4.18 BLI Analysis

The BLI assay was performed using a ForteBio Octet R8 system (Sartorius). The ZAP‐S‐His‐tag protein was precoated onto nickel‐nitrilotriacetic acid (Ni‐NTA) biosensors. A gradient concentration of compound **7** was diluted and added to the biosensors. The background binding controls used a duplicate set of sensors incubated in a phosphate‐buffered saline (PBS) buffer. The association and dissociation periods were set to 60 s. The assays were conducted in 96‐well black microplates (Greiner Bio‐One, 655209) with each well containing a total volume of 200 µL. Data were analyzed utilizing Octet data analysis software.

### 4.19 Plasmids and siRNA

The ZAP‐S sequence containing KpnI and EcoRI restriction sites was amplified from H1299 cells and inserted into pcDNA4.0. The mutation plasmids were constructed with a Mut Express II fast Mutagenesis Kit V2 kit (Vazyme, C214) using ZAP‐S plasmids as a template. The siRNA (5'‐CGATGAGTAAGATGCTTGTTAAGCA‐3' and 5'‐CGAUGAGUAAGAUGCUUGUUAAGCA‐3') targeted ZAP‐S obtained from the literature [33] and was synthesized by Beijing Tsingke Biotech (Beijing, China). For transfection, the cells were cultured for 24 h, and the plasmids or siRNA were transfected into cells using Lipofectamine 3000 (ThermoFisher Scientific, L3000015) and Lipofectamine RNAiMAX reagent (ThermoFisher Scientific, 13778). The transfected cells were incubated for 24 h before further treatment. Primers used in this study are detailed in Table S6.

### 4.20 Statistical Analysis

Student's *t*‐test was used to compare values between the two groups. Multiple‐group comparisons were conducted using either a one‐way or two‐way analysis of variance (ANOVA). GraphPad Prism 8 (GraphPad Inc., La Jolla, CA, USA) was used for statistical analysis. All of the data are expressed as mean ± standard deviation (SD). *p < 0.05, **p < 0.01, ***p < 0.001, ****p < 0.0001.

## Author Contributions

Xiaozhong Peng, Xiandao Pan, and Shuaiyao Lu conceived the idea and designed the experiment. Tingfu Du, Ruixue Liu, Xintian Zhang, and Longying Shen performed the main experiments, analyzed the data, and co‐wrote the main manuscript. Cong Tang, Junbin Wang, Yu Cheng, Wenhai Yu, and Bin Yin participated in this work. Xiaozhong Peng, Xiandao Pan, and Shuaiyao Lu revised the manuscript. All authors have read and approved the final manuscript.

## Funding

This work was supported by the Major Program of the National Natural Science Foundation of China (82394462), the Foundation for Innovative Research Groups of the National Natural Science Foundation of China (82221004), Chinese Academy of Medical Sciences (CAMS) Innovation Fund for Medical Sciences (2023‐I2M‐2‐001, 2021‐I2M‐1‐024, 2022‐I2M‐2‐002), State Key Laboratory Special Fund 2060204 and the Innovation project of the Academy of Medical Sciences (2022‐I2M‐CoV19‐001).

## Ethics Statement

All infection experiments involving SARS‐CoV‐2 were carried out at the National Kunming High‐level Biosafety Primate Research Center and the Biosafety Level 3 Laboratory of the Institute of Laboratory Animal Science, adhering to the standard operating procedures for Biosafety Level 3. The animal experiments were approved by the Experimental Animal Ethics Committee of the Institute of Medical Biology, Chinese Academy of Medical Sciences (approval number: DWSP202109002).

## Conflicts of Interest

The authors declare no conflicts of interest.

## Supporting information




**Figure S1**. Details of each compound.
**Figure S2**. Compound 7 reversed SARS‐CoV‐2‐induced changes in host gene expression.
**Figure S3**. Compound 7 reduced pathological damage and viral content in the lung tissue of Omicron‐infected hamster models.
**Figure S4**. The fold changes of F/R after treatment with drugs in SARS and MERS.
**Figure S5**. Molecular docking results for ZAP with lycorine.
**Table S1**. The CC_50_ values of lycorine and compound 7 in Huh‐7, H1299, and Cp‐H209
**Table S3**. The compound 7 potential targets identified by LC/MS‐MS.
**Table S4**. Atomic distance and binding energy of molecular interaction.
**Table S5**. Primers used in this study.
**Table S6**. Sequences of primers used for plasmid constructions.

**Supporting Information file S1**: mco270715‐sup‐0002‐tableS2.xls

## Data Availability

All data are available in the main text or the supplementary materials. Raw data are available from Mendeley Data at https://data.mendeley.com/datasets/zm3×33hvs2/1. Raw RNA‐seq data files are available at the National Genomics Data Center (NGDC), China National Center for Bioinformation, under the accession number HRA004067 (https://ngdc.cncb.ac.cn/gsa‐human/browse/HRA004067). The materials during the current study are available from the corresponding author on reasonable request.
