# Correction to “Lycorine Derivative Inhibits SARS‐CoV‐2 Replication by Reducing −1 Programmed Ribosomal Frameshifting via Targeting ZAP”

**DOI:** 10.1002/mco2.70757

**Published:** 2026-05-29

Du T, Liu R, Zhang X, Shen L, Tang C, Wang J, Cheng Y, Yu W, Yin B, Lu S, Pan X, Peng X. Lycorine Derivative Inhibits SARS‐CoV‐2 Replication by Reducing −1 Programmed Ribosomal Frameshifting via Targeting ZAP. *MedComm*. 2026; DOI: 10.1002/mco2.70715

In the originally published version of this article, the statement indicating equal contribution among authors appeared incorrectly on the first page. The original statement read:

“Tingfu Du1, Ruixue Liu, Xintian Zhang these authors contributed equally to this work.”

This statement should have read:

“Tingfu Du, Ruixue Liu, Xintian Zhang and Longying Shen contributed equally to this work.”

We apologize for this error.

