# Supplementary material for: Lycorine Derivative Inhibits SARS‐CoV‐2 Replication by Reducing −1 Programmed Ribosomal Frameshifting via Targeting ZAP
Source: MedComm (2020). 2026 Apr 6;7(4):e70715. doi: 10.1002/mco2.70715 (PMC13053664; doi:10.1002/mco2.70715)
Supplement: Supplementary file 1 — Figure S1. Details of each compound. Figure S2. Compound 7 reversed SARS‐CoV‐2‐induced changes in host gene expression. Figure S3. Compound 7 reduced pathological damage and viral content in the lung tissue of Omicron‐infected hamster models. Figure S4. The fold changes of F/R after treatment with drugs in SARS and MERS. Figure S5. Molecular docking results for ZAP with lycorine. Table S1. The CC50 values of lycorine and compound 7 in Huh‐7, H1299, and Cp‐H209 Table S3. The compound 7 potential targets identified by LC/MS‐MS. Table S4. Atomic distance and binding energy of molecular interaction. Table S5. Primers used in this study. Table S6. Sequences of primers used for plasmid constructions. [file MCO2-7-e70715-s002.docx]

**Lycorine derivative inhibits SARS-CoV-2 replication by reducing −1 programmed ribosomal frameshifting via targeting ZAP**

**Figure S1. Details of each compound.**

**
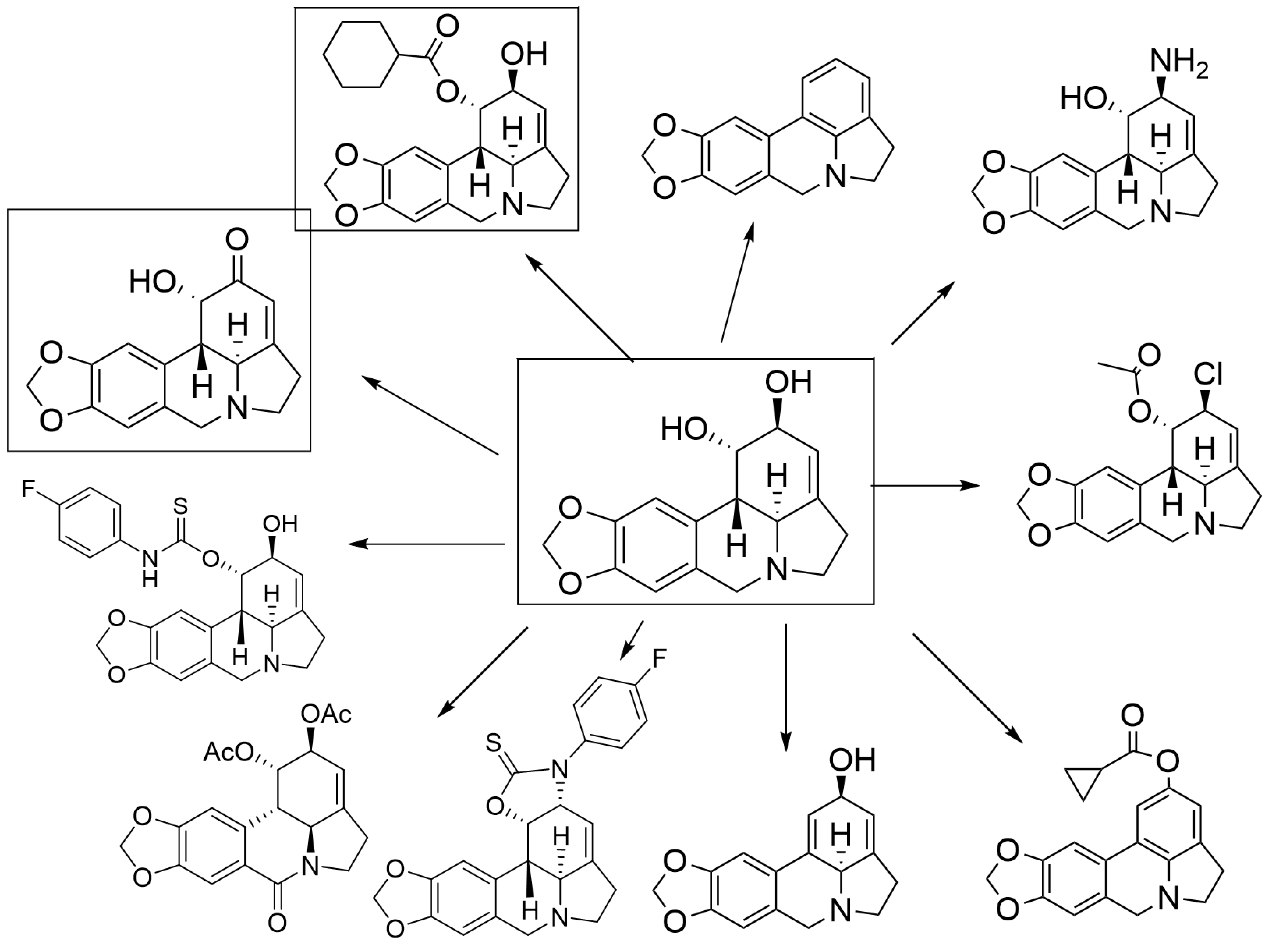
**

The structural diversity of derivatives of lycorine.

All chemicals were purchased from Innochem, Aladdin, Adamas, Alfa, or other chemical companies. All chemicals were used without further purification unless otherwise mentioned. Lycorine was purchased from Xi'an Green Biotechnology Co., Ltd. with a purity of over 98%. Thin layer chromatography (TLC) was run on silica gel plates (GF254) from Qingdao Haiyang. Biotage automatic column chromatography machine was made by Biotage with Isolera One. Nuclear magnetic resonance (NMR) was performed on Bruker AV 400 or 500 MHz (Bruker Company, USA) with tetramethylsilane as internal reference. Mass chromatography was performed on Waters ACQUITY UPLC H-Glass with an ESI source，high resolution mass chromatography was performed on Thermo Scientific LC-ESI-MS Exactive Plus.

**3**

White solid; yield 50%. ^1^H NMR (500 MHz, CDCl_3_) δ 6.60 (s, 1H), 6.55 (s, 1H), 5.90 (s, 2H), 5.56 (s, 1H), 5.51 (s, 1H), 4.33-4.01 (m, 2H), 2.84 (d, *J* = 10.4 Hz, 1H), 2.75 (d, *J* = 10.4 Hz, 1H), 2.60 (s, 2H), 2.40-2.29 (m, 1H), 1.91 (s, 3H). HR-MS(ESI) m/z calcd for C_18_H_20_NO_5_ [M+H]^+^ 330.1336, found 330.1333.

**4**

White solid; yield 45%. ^1^H NMR (400 MHz, CDCl_3_) δ 6.62 (s, 1H), 6.56 (s, 1H), 5.88 (s, 2H), 5.58 (s, 1H), 5.53 (s, 1H), 4.12 (d, *J* = 15.0 Hz, 2H), 3.53 (s, 1H), 3.32 (s, 1H), 2.94-2.30 (m, 7H), 1.00 (d, *J* = 7.0 Hz, 3H), 0.94 (d, *J* = 7.0 Hz, 3H). ^13^C NMR (101 MHz, CDCl_3_) δ 176.88, 146.88, 146.27, 140.75, 129.32, 117.85, 107.21, 105.03, 100.98, 72.27, 69.43, 62.17, 56.92, 53.79, 39.18, 33.94, 28.58, 18.97, 18.79. ESI-MS: 358 [M+H]^+^

**5**

White solid; yield 33%. ^1^H NMR (400 MHz, CDCl_3_) δ 6.59 (s, 1H), 6.56 (s, 1H), 5.90 (q, *J* = 1.5 Hz, 2H), 5.63 (s, 1H), 5.53 (s, 1H), 4.19-4.13 (m, 2H), 3.61-3.48 (m, 1H), 3.38-3.29 (m, 1H), 3.21-2.54 (m, 10H), 2.50-2.30 (brs, 1H). ^13^C NMR (101 MHz, CDCl_3_) δ 175.35, 146.53, 146.25, 143.96, 129.44, 126.80, 117.47, 107.36, 105.11, 101.03, 72.50, 69.79, 61.74, 56.99, 53.84, 39.64, 38.12, 28.77, 25.24, 18.53. ESI-MS: 370 [M+H]^+^

**6**

White solid; yield 31%. ^1^H NMR (400 MHz, CDCl_3_) δ 6.62 (s, 1H), 6.55 (s, 1H), 5.88 (s, 2H), 5.58 (s, 1H), 5.53 (s, 1H), 4.13 (s, 2H), 3.49 (s, 1H), 3.32 (s, 1H), 3.10-2.23 (m, 7H), 1.76-1.37 (m, 8H).^13^C NMR (101 MHz, CDCl_3_) δ 176.52, 146.53, 146.24, 144.33, 126.78, 117.05, 107.36, 105.13, 101.03, 72.49, 69.77, 61.82, 57.01, 53.87, 43.88, 39.63, 30.05, 29.86, 28.79, 25.64. ESI-MS: 384 [M+H]^+^

**7**

White solid; yield 25%. ^1^H NMR (400 MHz, CDCl_3_) δ 6.58 (s, 1H), 6.54 (s, 1H), 5.87 (d, *J* = 1.5 Hz, 2H), 5.56 (s, 1H), 5.49 (s, 1H), 4.24-3.55 (m, 3H), 3.48 (d, *J* = 13.9 Hz, 1H), 3.31 (dt, *J* = 9.2, 4.8 Hz, 1H), 2.84 (d, *J* = 10.3 Hz, 1H), 2.73 (d, *J* = 10.5 Hz, 1H), 2.65-2.55 (m, 2H), 2.38 (t, *J* = 8.9 Hz, 1H), 2.13 (tt, *J* = 11.0, 3.6 Hz, 1H), 1.73-1.43 (m, 5H), 1.36-1.01 (m, 5H). ^13^C NMR (101 MHz, CDCl_3_) δ 175.77, 146.46, 146.15, 143.34, 129.23, 127.29, 117.65, 107.29, 105.07, 100.95, 72.26, 69.47, 61.76, 56.88, 53.79, 42.88, 39.34, 28.81, 28.77, 28.68, 25.69, 25.22. HR-MS(ESI) m/z calcd for C_23_H_28_NO_5_ [M+H]^+^ 398.1962, found 398.1971.

**8**

White solid; yield 33%. ^1^H NMR (400 MHz, CDCl3) δ 6.60 (s, 1H), 6.57 (s, 1H), 5.90 (s, 2H), 5.64 (s, 1H), 5.54 (s, 1H), 4.16 (s, 2H), 3.58-3.45 (m, 1H), 3.42 – 3.32 (m, 1H), 3.04 – 2.29 (m, 11H). ^13^C NMR (101 MHz, CDCl3) δ 203.70, 173.25, 146.58, 143.41, 129.32, 126.32, 117.54, 107.34, 105.43, 101.01, 98.65, 73.84, 72.78, 69.36, 61.68, 57.60, 51.99, 39.22, 36.22, 28.62. ESI-MS: 384 [M+H]^+^ for C_21_H_22_NO_6_.

**9**

White solid; yield 31%. ^1^H NMR (400 MHz, CDCl_3_) δ 6.64 (s, 1H), 6.56 (s, 1H), 5.90 (s, 2H), 5.60 (s, 1H), 5.53 (s, 1H), 4.20-4.08 (m, 2H), 3.58-3.47 (m, 1H), 3.40-3.29 (m, 1H), 3.18-2.89 (m, 2H), 2.87 (d, *J* = 10.1 Hz, 1H), 2.76 (s, 1H), 2.66-2.59 (m, 2H), 2.53-2.29 (brs, 1H), 2.14-2.05 (m, 2H), 2.05-1.98 (m, 2H), 1.90-1.72 (m, 2H). ^13^C NMR (101 MHz, CDCl_3_) δ 173.00, 146.59, 146.40, 143.81, 129.44, 126.74, 118.70 (dd, *J* = 282, 270 Hz), 117.35, 107.45, 104.81, 101.09, 73.44, 69.43, 61.74, 56.95, 53.81, 39.45, 38.68 (t, *J* = 24.4 Hz), 28.65, 26.55 (dd, *J* = 13.8, 6.2 Hz), 22.73. ESI-MS: 406 [M+H]^+^

**10**

White solid; yield 40%. ^1^H NMR (500 MHz, CDCl_3_) δ 6.68 (s, 1H), 6.58 (s, 1H), 6.00 (s, 2H), 5.91 (s, 2H), 4.17 (d, *J* = 13.8 Hz, 1H), 3.59 (d, *J* = 10.8 Hz, 1H), 3.47 (s, 1H), 3.31 (d, *J* = 10.3 Hz, 1H), 3.13 (d, *J* = 10.1 Hz, 1H), 3.02-2.39 (m, 11H). ^13^C NMR (101 MHz, CDCl_3_) δ 173.43, 146.55, 146.35, 143.60, 129.35, 126.98, 117.41, 107.39, 105.00, 101.05, 76.60, 73.15, 69.47, 69.32, 61.75, 56.97, 53.79, 39.44, 30.57, 28.70, 24.78, 22.74. ESI-MS: 386 [M+H]^+^

**11**

White solid; yield 45%. ^1^H NMR (400 MHz, CDCl_3_) δ 6.80 (t, *J* = 8.7 Hz, 2H), 6.69-6.48 (m, 4H), 5.93 (d, *J* = 12.2 Hz, 2H), 5.67 (s, 1H), 5.50 (s, 1H), 4.47 (s, 2H), 4.22-4.04 (m, 2H), 3.61-2.16 (m, 8H). ^13^C NMR (101 MHz, CDCl_3_) δ 168.69, 157.66 (d, *J* = 239.1 Hz), 153.68 (d, *J* = 2.2 Hz), 146.73, 146.53, 143.74, 129.17, 126.49, 117.32, 115.91 (d, *J* = 23.2 Hz), 115.44 (d, *J* = 8.1 Hz), 107.57, 104.81, 101.20, 73.39, 69.23, 65.60, 61.49, 56.69, 53.78, 39.13, 28.64. ESI-MS: 440 [M+H]^+^

**12**

White solid; yield 41%. ^1^H NMR (400 MHz, CDCl_3_) δ 7.05 (d, *J* = 8.5 Hz, 2H), 6.64-6.53 (m, 4H), 5.98 (s, 1H), 5.93 (s, 1H), 5.72 (s, 1H), 5.53 (s, 1H), 4.51 (s, 2H), 4.19 (s, 1H), 4.10 (d, *J* = 14.1 Hz, 1H), 3.41-3.32 (m, 2H), 2.88 (d, *J* = 10.4 Hz, 1H), 2.73-2.03 (m, 7H). ESI-MS: 456 [M+H]^+^

**13**

White solid; yield 62%. ^1^H NMR (500 MHz, Chloroform-*d*) δ 7.20 – 7.12 (m, 2H), 6.91 – 6.83 (m, 2H), 6.54 (d, *J* = 4.3 Hz, 2H), 5.90 (dd, *J* = 7.5, 1.4 Hz, 2H), 5.57 (s, 1H), 5.45 (s, 1H), 4.11 (d, *J* = 14.0 Hz, 1H), 4.06 – 4.01 (m, 1H), 3.48 – 3.36 (m, 3H), 3.32 (dt, *J* = 9.2, 4.7 Hz, 1H), 2.80 (d, *J* = 10.4 Hz, 1H), 2.61 – 2.52 (m, 3H), 2.31 (q, *J* = 8.9 Hz, 1H). ^13^C NMR (126 MHz, CDCl_3_) δ 169.52, 163.25, 161.29, 146.63, 146.47, 143.94, 132.85, 132.78, 129.66, 129.64, 129.37, 126.72, 117.34, 116.30, 116.13, 107.46, 105.06, 101.15, 73.78, 69.40, 61.63, 56.95, 53.79, 39.39, 37.42, 28.65; HR-MS(ESI) m/z calcd for C_24_H_23_NFSO_5_ [M+H]^+^ 456.1276, found 456.1274.

**14**

White solid; yield 25%. ^1^H NMR (400 MHz, CDCl_3_) δ 7.16-7.04 (m, 2H), 6.86 (t, *J* = 8.5 Hz, 2H), 6.65 (s, 1H), 6.56 (s, 1H), 5.90 (d, *J* = 3.3 Hz, 2H), 5.62 (s, 1H), 5.53 (s, 1H), 4.12 (d, *J* = 14.1 Hz, 2H), 3.60-3.27 (m, 3H), 2.85 (s, 1H), 2.68-2.17 (m, 5H), 1.28 (d, *J* = 7.2 Hz, 3H). ^13^C NMR (101 MHz, CDCl_3_) δ 172.35, 162.74 (d, *J* = 248.3 Hz), 146.66, 146.44, 143.94, 135.29 (d, *J* = 8.3 Hz), 129.15, 127.90, 127.87, 126.79, 117.44, 116.01 (d, *J* = 22.0 Hz), 107.46, 105.15, 101.11, 73.26, 69.50, 61.60, 56.76, 53.77, 45.18, 39.37, 28.72, 17.37. HR-MS(ESI) m/z calcd for C_25_H_25_NFSO_5_ [M+H]^+^ 470.1432, found 470.1436.

General synthesis procedures of lycorine derivatives (**15**-**17**)

**2**

Lycorine (1 mmol) was dissolved in 3 ml of anhydrous DMF and pyridine (200 μ l. 2.5 mmol), then Dess- Martin periodinane reagent (1.1 mmol) was added in batches and stirred at room temperature. After lycorine was reacted over, the reactants are concentrated, diluted with water, the aqueous solution was extracted with DCM for three times, then organic phase was washed with brine, dried with anhydrous Na_2_SO_4_, and concentrated to obtain **2** as a light pink solid; 45%. ^1^H NMR (400 MHz, CDCl_3_) δ 6.76 (s, 1H), 6.60 (s, 1H), 5.95 (d, *J* = 16.7 Hz, 3H), 4.55 (s, 1H), 4.17 (d, *J* = 14.0 Hz, 1H), 3.63 (d, *J* = 14.1 Hz, 1H), 3.46 (s, 1H), 3.31 (s, 1H), 3.14 (d, *J* = 11.4 Hz, 1H), 2.87 (d, *J* = 7.3 Hz, 2H), 2.49 (brs, 2H). ESI-MS: 286 [M+H]^+^

A solution of **2** (1 mmol), acids (1.2 mmol), EDCI (1.2 mmol), and DMAP (0.2 mmol) in DCM (5 ml) was stirred at room temperature for 4 h. Then, 30 ml DCM was added. The organic phase was washed with saturated NaHCO_3_ (20 ml), H_2_O (20 ml), and brine (20 ml). Dried over Na_2_SO_4_, the solvent was evaporated. The residue was separated by rapid silica gel column chromatography (eluent: PE-EtOAc) to get target compound **15**-**17**.

**15**

White solid; yield 41%. ^1^H NMR (400 MHz, CDCl_3_) δ 6.72 (s, 1H), 6.56 (s, 1H), 5.98 (s, 2H), 5.90 (s, 2H), 4.16 (d, *J* = 14.1 Hz, 1H), 3.58 (d, *J* = 14.1 Hz, 1H), 3.45 (dt, *J* = 9.1, 4.5 Hz, 1H), 3.26 (d, *J* = 11.7 Hz, 1H), 3.14 (d, *J* = 9.9 Hz, 1H), 2.98 (p, *J* = 8.3 Hz, 1H), 2.86 (s, 2H), 2.52 (q, *J* = 8.7 Hz, 1H), 2.16-2.05 (m, 2H), 2.03-1.93 (m, 2H), 1.89-1.75 (m, 2H). ^13^C NMR (101 MHz, CDCl_3_) δ 193.16, 174.04, 168.98, 146.60, 128.84, 125.29, 120.44, 107.27, 105.56, 101.08, 68.65, 62.42, 56.33, 53.24, 45.56, 37.79, 30.00, 25.04, 18.41. ESI-MS: 368 [M+H]^+^

**16**

White solid; yield 40%. ^1^H NMR (400 MHz, CDCl_3_) δ 6.70 (s, 1H), 6.56 (s, 1H), 5.98 (s, 2H), 5.90 (s, 2H), 4.16 (d, *J* = 14.1 Hz, 1H), 3.59 (d, *J* = 14.1 Hz, 1H), 3.45 (dt, *J* = 9.1, 4.5 Hz, 1H), 3.26 (d, *J* = 10.0 Hz, 1H), 3.16 (d, *J* = 9.7 Hz, 1H), 2.86 (s, 2H), 2.65-2.47 (m, 2H), 1.74-1.64 (m, 2H), 1.65-1.40 (m, 6H). ^13^C NMR (101 MHz, CDCl_3_) δ 193.27, 175.26, 169.44, 146.70, 128.93, 125.44, 120.57, 107.37, 105.72, 101.18, 68.73, 62.62, 56.46, 53.37, 45.69, 43.62, 30.12, 30.02, 29.58, 25.65, 25.59. ESI-MS: 382 [M+H]^+^

**17**

White solid; yield 45%. ^1^H NMR (400 MHz, CDCl_3_) δ 6.70 (s, 1H), 6.56 (s, 1H), 5.98 (s, 2H), 5.90 (s, 2H), 4.17 (d, *J* = 14.1 Hz, 1H), 3.60 (d, *J* = 14.1 Hz, 1H), 3.54-3.39 (m, 1H), 3.27 (d, *J* = 10.2 Hz, 1H), 3.17 (d, *J* = 9.9 Hz, 1H), 2.86 (s, 2H), 2.53 (q, *J* = 8.8, 8.3 Hz, 1H), 2.23-2.11 (m, 1H), 1.78-1.43 (m, 7H), 1.38-1.20 (m, 3H). ^13^C NMR (101 MHz, CDCl_3_) δ 193.25, 174.50, 168.93, 146.72, 128.89, 125.42, 120.60, 107.38, 105.82, 101.20, 68.58, 62.61, 56.44, 53.37, 45.66, 42.85, 30.12, 28.85, 28.69, 25.74, 25.24, 25.18. HR-MS(ESI) m/z calcd for C_23_H_26_NO_5_ [M+H]^+^ 396.1806, found 396.1810.

**Figure S2.** **Compound 7 reversed SARS-CoV-2 induced changes in host gene expression.**

Total RNA was extracted from Calu-3 and Huh-7 cells treated with original SARS-CoV-2 (MOI = 0.2) and compound **7** (10 μM) for 48 hours. RNA quality was evaluated on an Agilent 2100 Bioanalyzer (Agilent Technologies, Palo Alto, CA, USA). cDNA library sequencing was conducted on the Illumina Hiseq2500 by Gene Denovo Biotechnology Co., Ltd (Guangzhou, China). DESeq2 software was used to analyze RNA differential expression between the two groups. Genes with false discovery rate (FDR)≤ 0.05 and absolute fold change ≥ 2 were considered as differentially expressed genes (DEGs). KEGG pathway enrichment analysis showed that signal transduction pathways in DEGs were significantly enriched compared with the whole-genome background. The calculated p-values were corrected by FDR with FDR ≤ 0.05 as a threshold. The pathways that satisfy this condition were defined as significantly enriched pathways in DEGs. RNA-seq analysis of infected Calu-3 and Huh-7 cells treated with compound **7** revealed a significant reversal of host gene expression changes induced by SARS-CoV-2.

(1) Cluster analysis of genes with transcriptional changes caused by SARS-CoV-2 infection and reversed by compound **7** treatment. Enriched sets of genes with expression significantly up-regulated by viral infection but down-regulated by compound **7** treatment or vice versa were plotted. Differential expression for each gene was determined by absolute fold change ≥ 2 with the parameter of FDR ≤0.05.


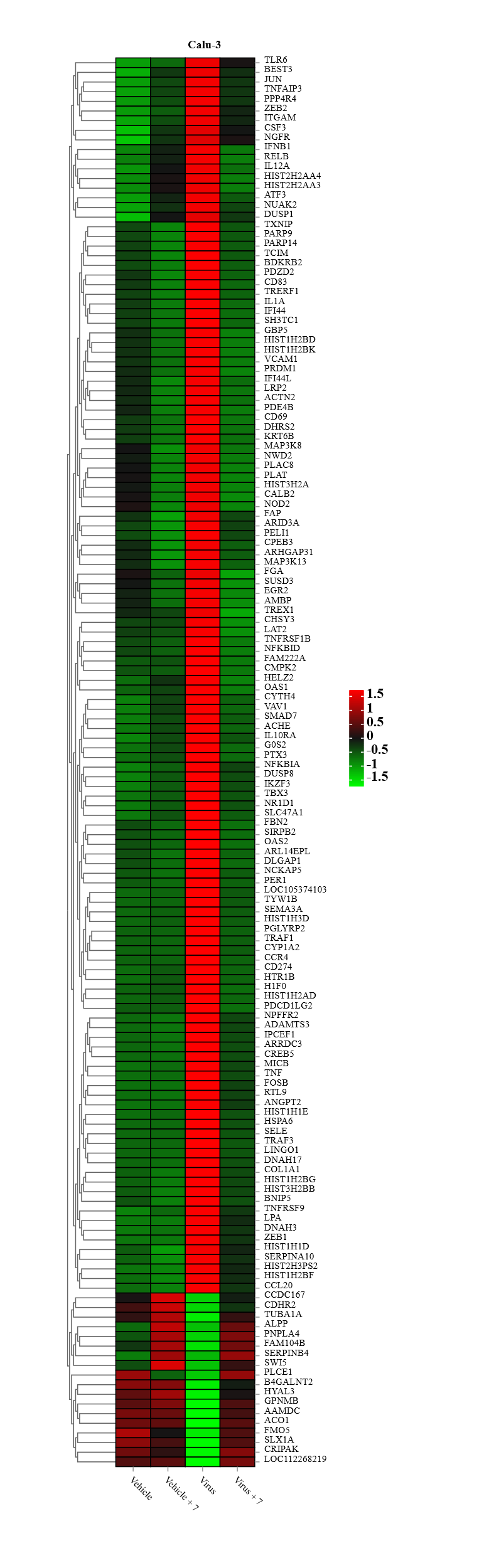

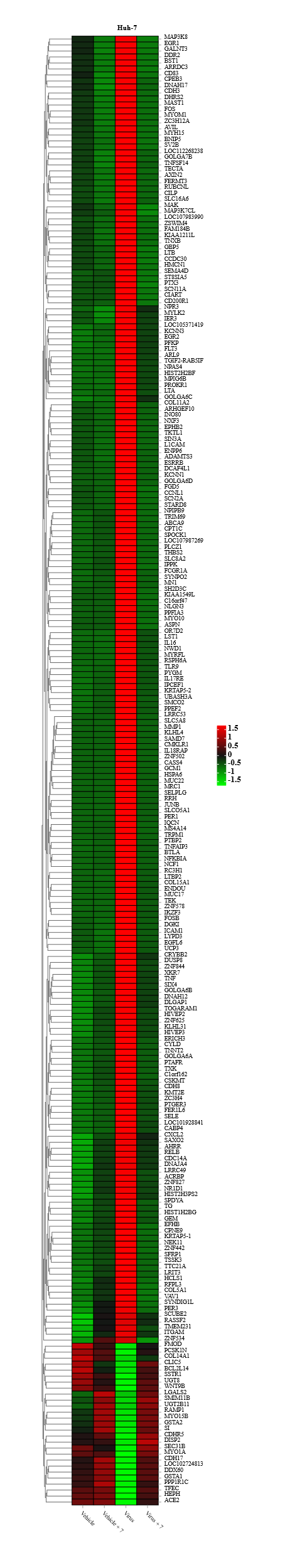


(2) A. Top 6 signaling pathways after infection by SARS-CoV-2 (GD108) in Huh-7 and Calu-3 cells, respectively. B. 6 signaling pathways after virus infection which were reversed after treatment with compound **7**. C. Heatmap for the selected genes from top 6 signaling pathways after virus infection which were reversed after treatment with compound **7**. data represent the log2FC values. n = 3 per group. D. Among these reversed genes, six genes (*RELB*, *NFKBIA*, *MAP3K8*, *TNF*, *TNFAIP3*, and *SELE*) were significantly upregulated in both Calu-3 and Huh-7 cells after virus infection and downregulated after treatment with compound **7**. These six genes are mainly related to the immune response after virus infection. This result was confirmed by real-time PCR analysis; n = 3 per group; ***p* < 0.01, ****p* < 0.001, *****p* < 0.0001.


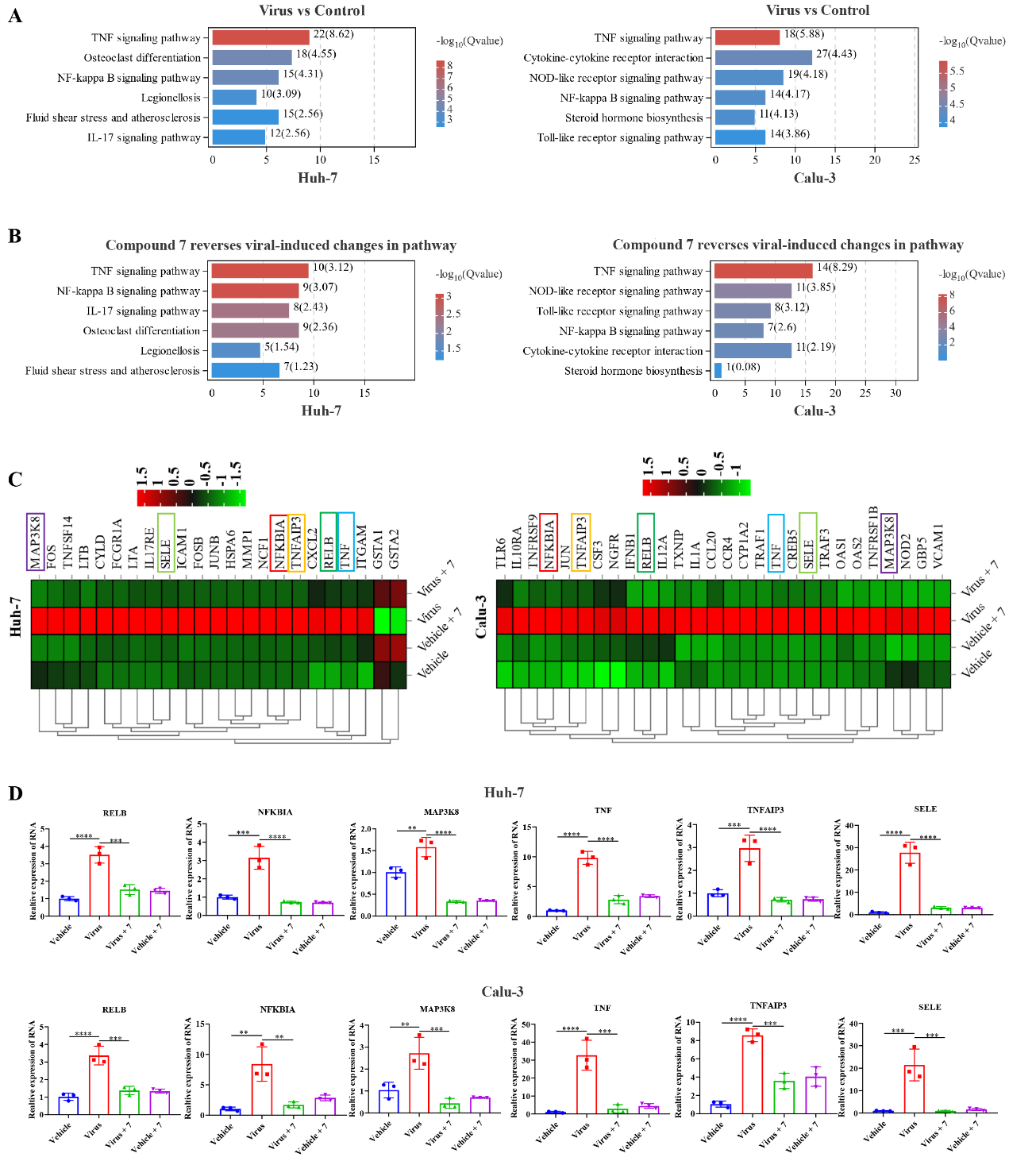


(3) Changes in the host NF-κB pathway following SARS-CoV-2 infection compared to uninfected Huh-7 cells. Up-regulated genes are colored red and down-regulated genes are colored green. Genes without significant differential expression are colored gray.


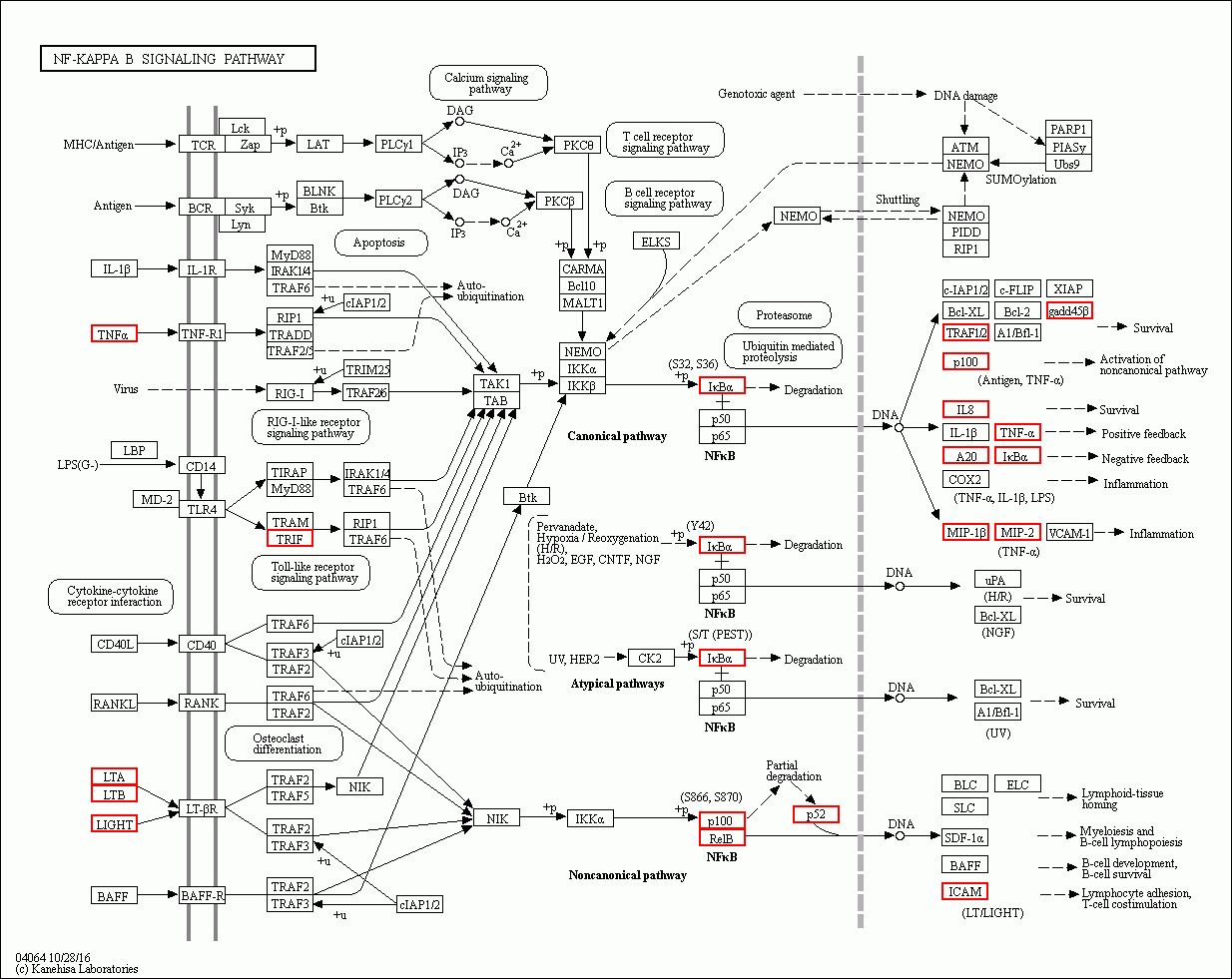


(4) Changes in the host NF-κB pathway following SARS-CoV-2 infection and reversed by compound **7** treatment in Huh-7 cells. Up-regulated genes are colored red and down-regulated genes are colored green. Genes without significant differential expression are colored gray.


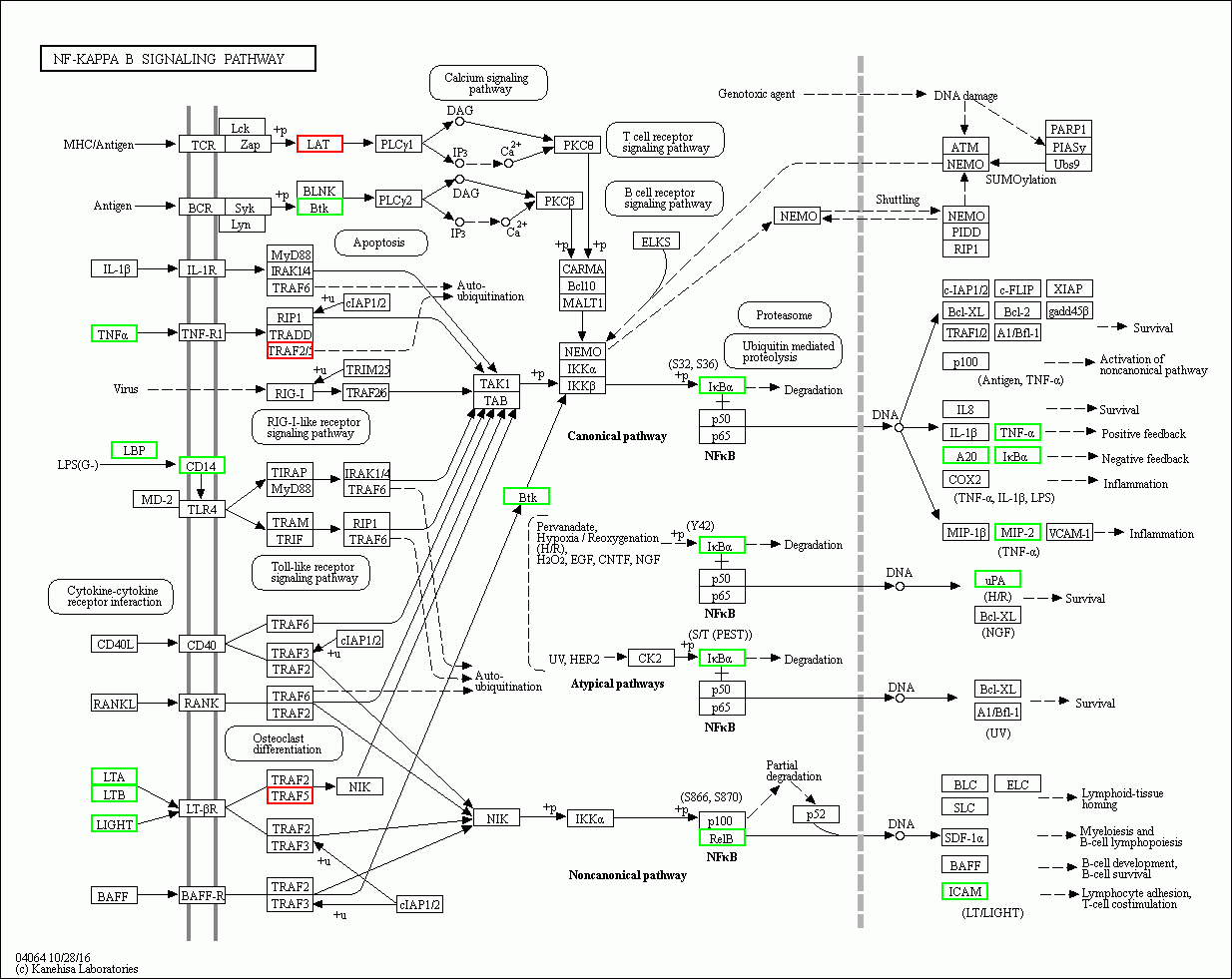


(5) Changes in the host TNF pathway following SARS-CoV-2 infection compared to uninfected Huh-7 cells. Up-regulated genes are colored red and down-regulated genes are colored green. Genes without significant differential expression are colored gray.


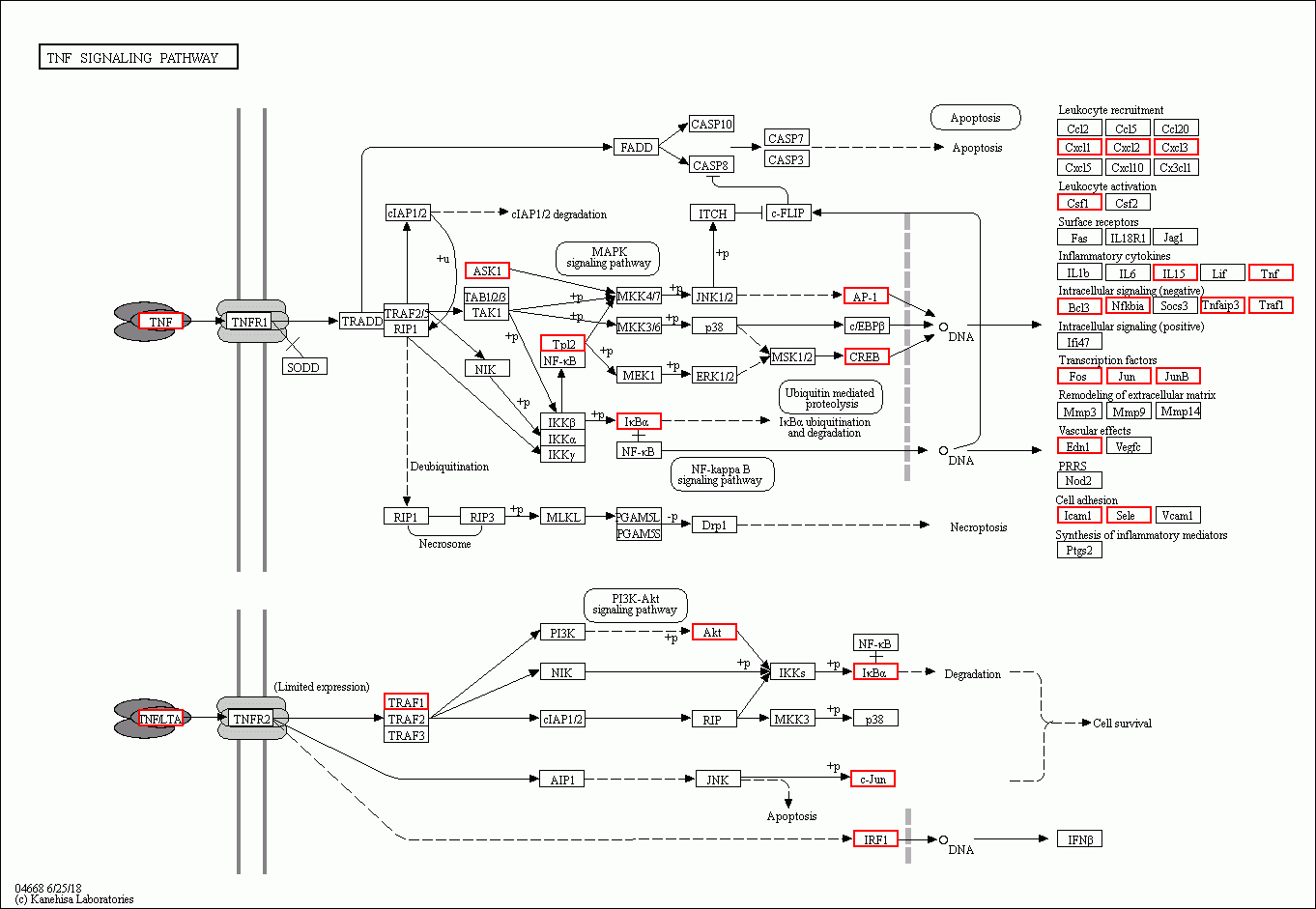


(6) Changes in the host TNF pathway following SARS-CoV-2 infection and reversed by compound **7** treatment in Huh-7 cells. Up-regulated genes are colored red and down-regulated genes are colored green. Genes without significant differential expression are colored gray.


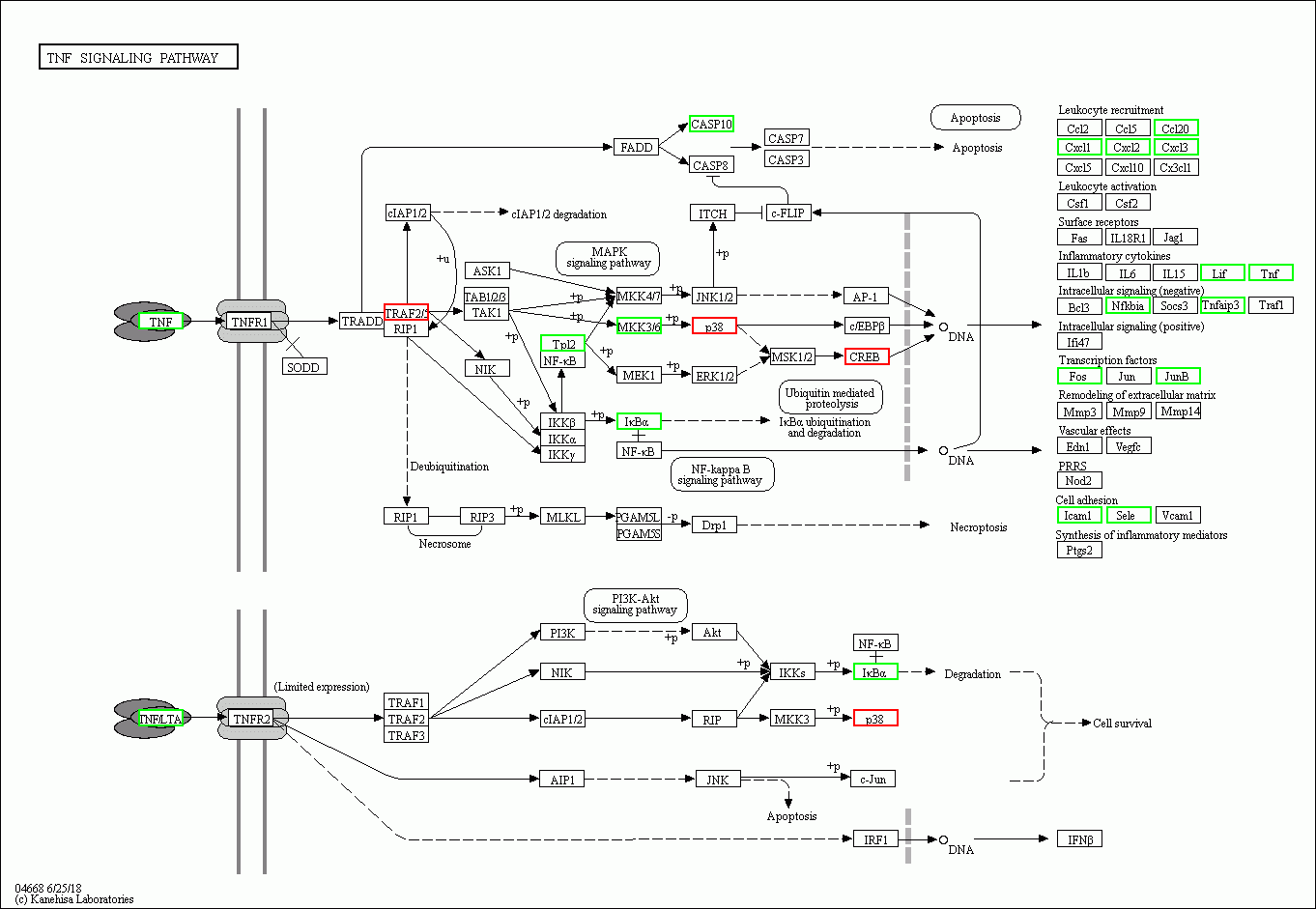


(7) Changes in the host NF-κB pathway following SARS-CoV-2 infection compared to uninfected Calu-3 cells. Up-regulated genes are colored red and down-regulated genes are colored green. Genes without significant differential expression are colored gray.


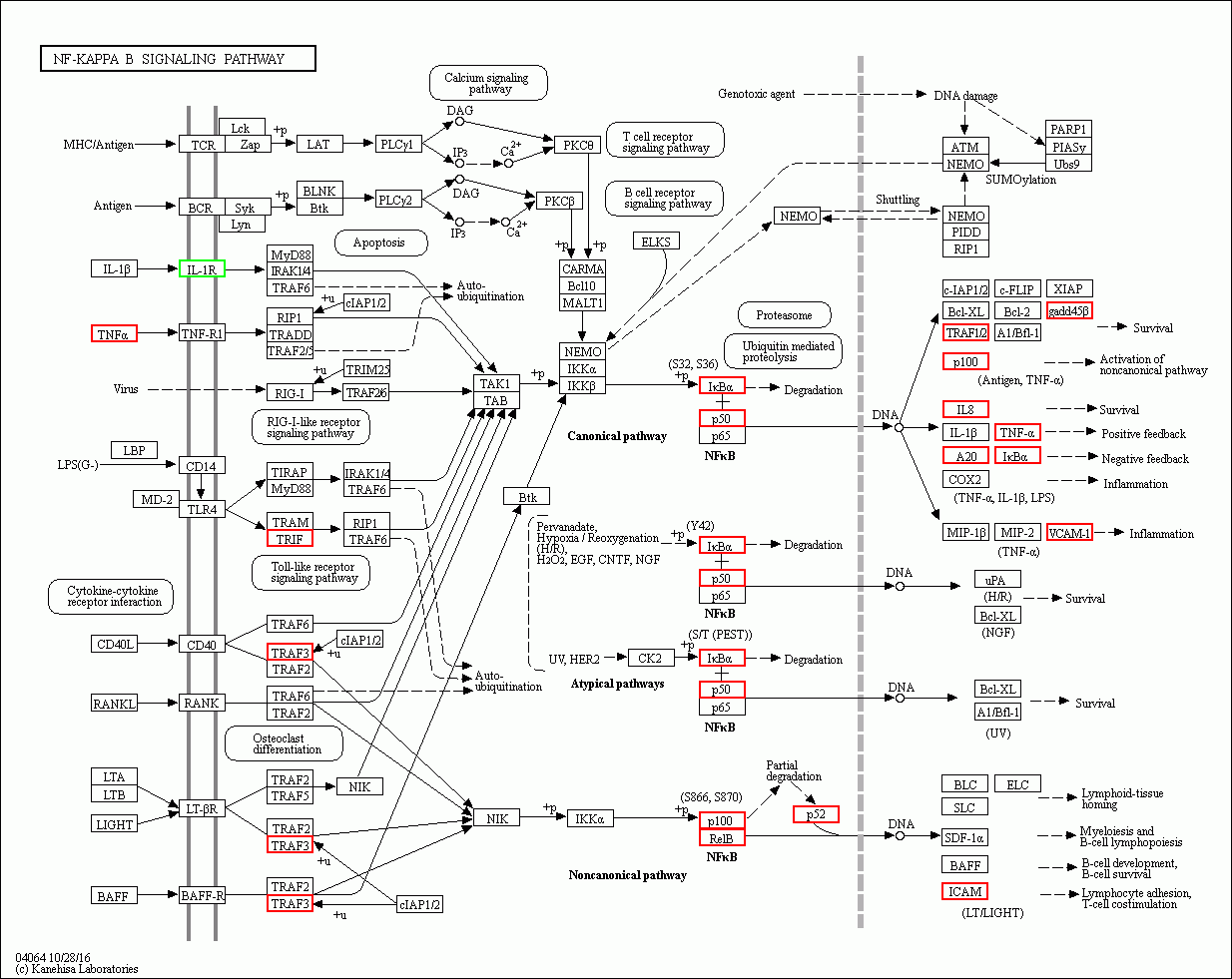


(8) Changes in the host NF-κB pathway following SARS-CoV-2 infection and reversed by compound **7** treatment in Calu-3. Up-regulated genes are colored red and down-regulated genes are colored green. Genes without significant differential expression are colored gray.


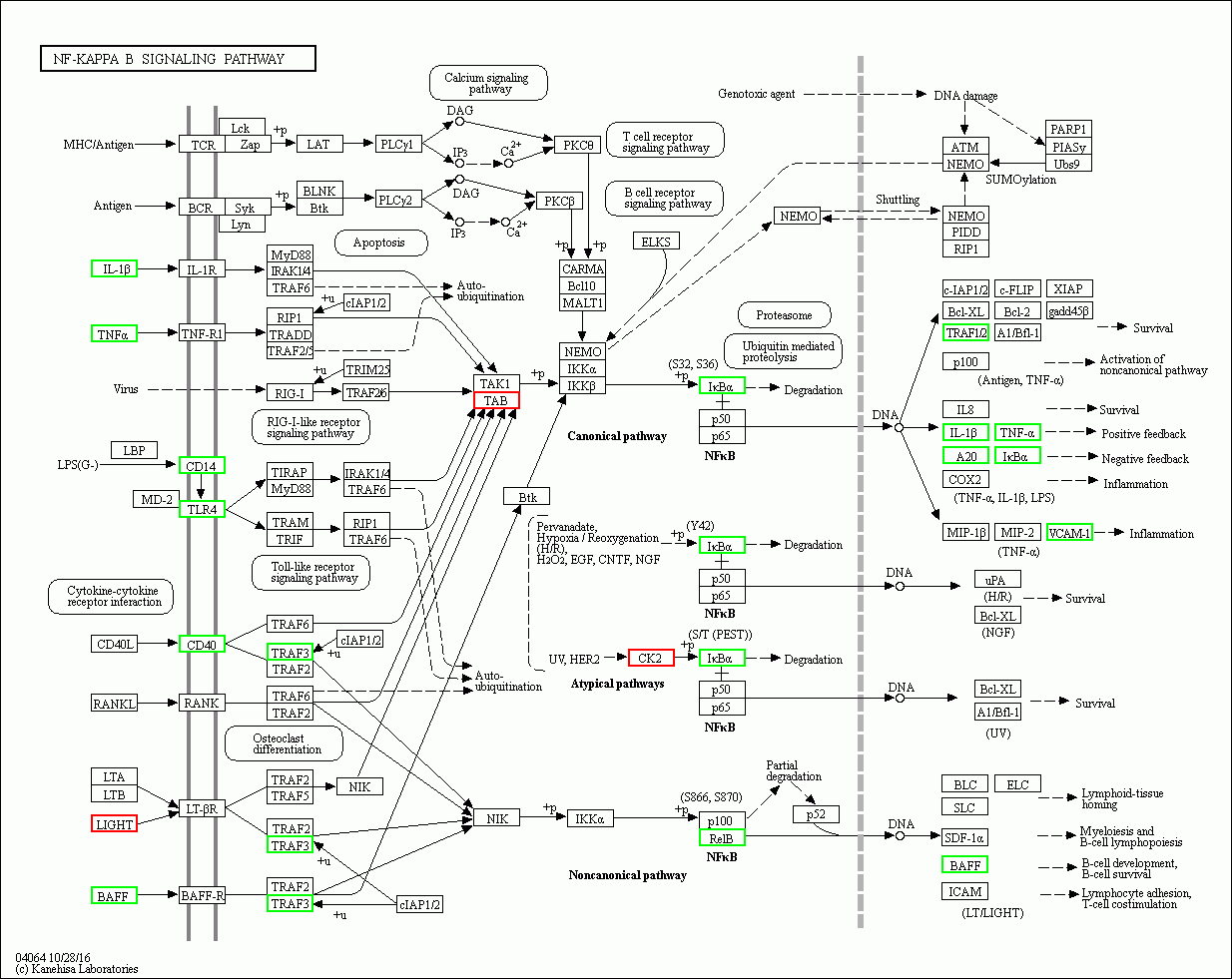


(9) Changes in the host TNF pathway following SARS-CoV-2 infection compared to uninfected Calu-3 cells. Up-regulated genes are colored red and down-regulated genes are colored green. Genes without significant differential expression are colored gray.


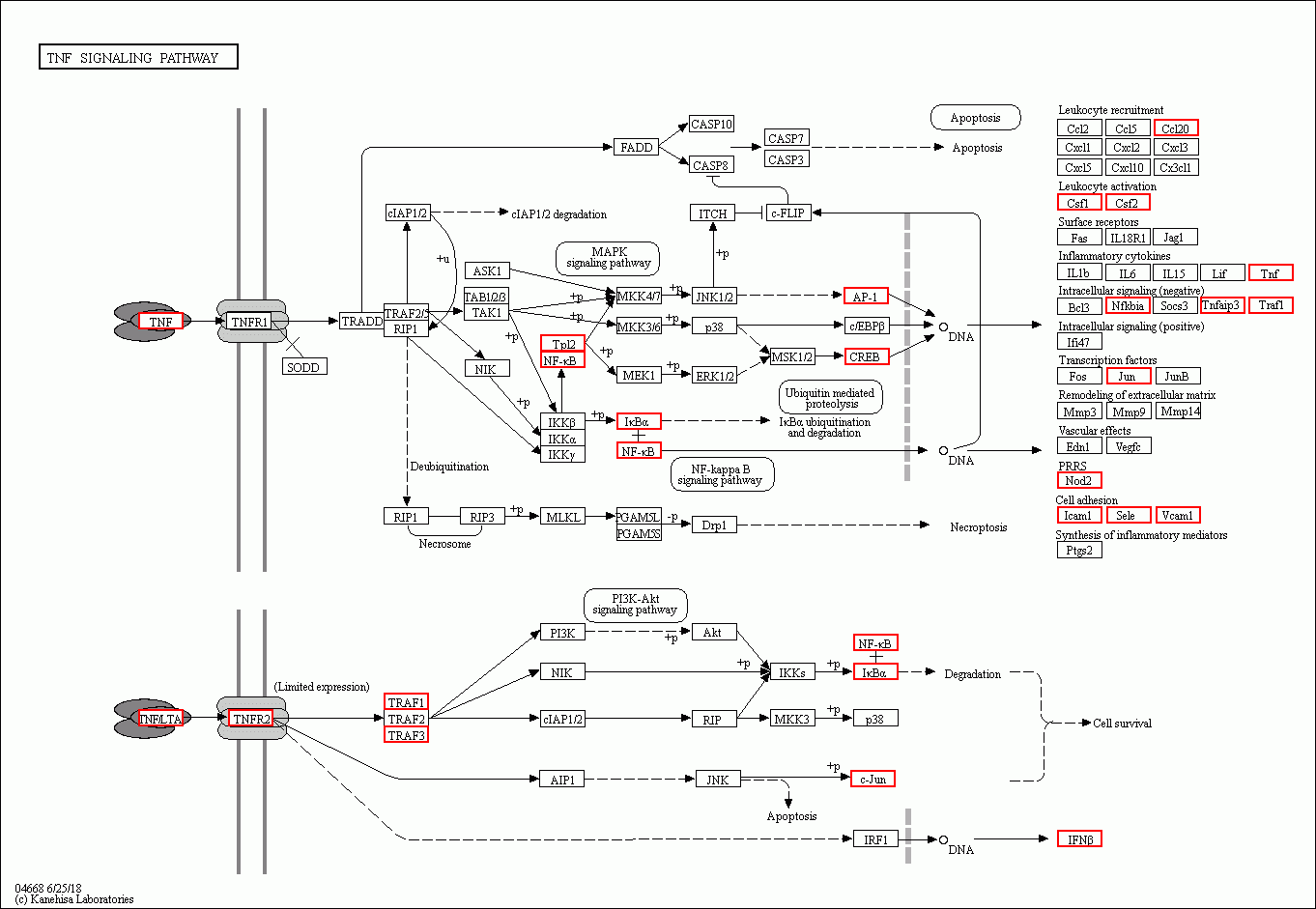


(10) Changes in the host TNF pathway following SARS-CoV-2 infection and reversed by compound **7** treatment in Calu-3. Up-regulated genes are colored red and down-regulated genes are colored green. Genes without significant differential expression are colored gray.


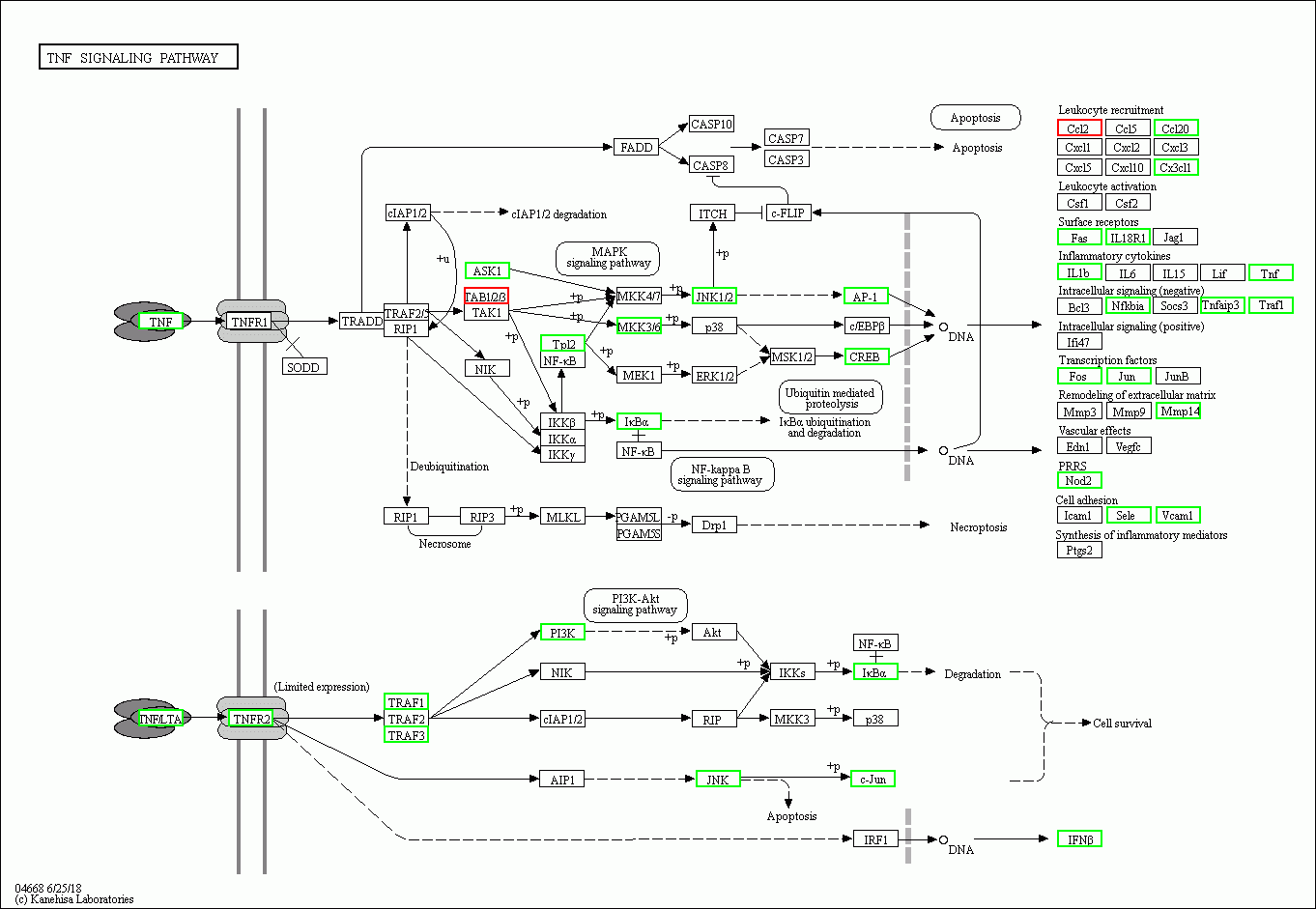


**Figure S3. Compound 7 reduced pathological damage and viral content in lung tissue of Omicron-infected hamster models.**


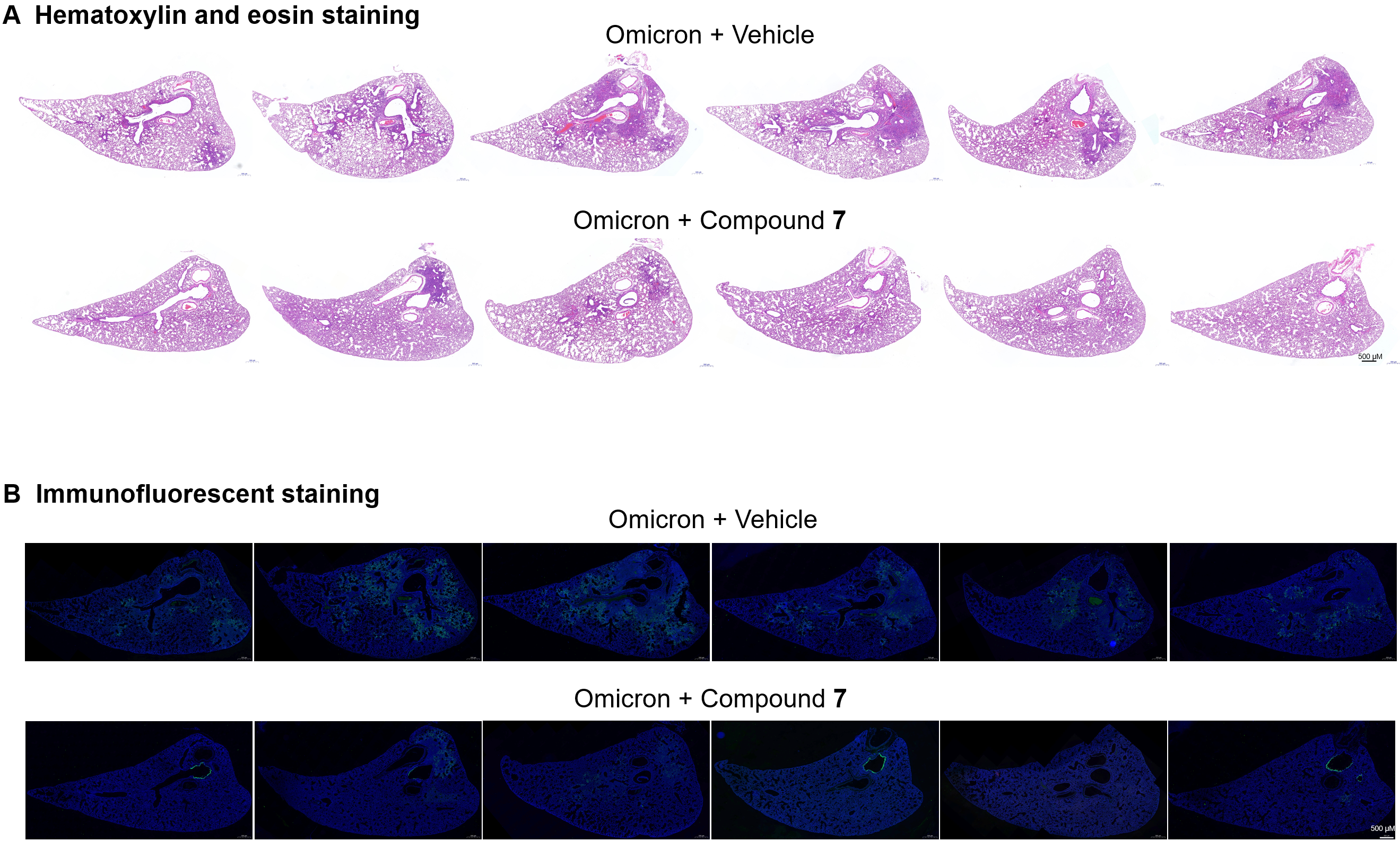


**A.** Hematoxylin and eosin (H&E) staining. H&E staining of lung tissue from infected hamster harvested at day 3 post infection. The top panel (Omicron + vehicle) shows lung tissue from vehicle-treated infected hamsters, exhibiting significant pathological damage, including inflammatory cell infiltration, alveolar wall thickening, and congestion. The bottom panel (Omicron + compound **7**) shows lung tissue from infected hamsters treated with compound **7**, demonstrating a marked reduction in pathological lesions and improved pulmonary architecture. Images are representative of n = 6 animals per group. **B.** Immunofluorescent staining. The top panel (Omicron + vehicle) shows abundant N protein signal (green) in the lungs of vehicle-treated infected hamsters, indicating high viral load and extensive infection. The bottom panel (Omicron + compound **7**) shows a substantial reduction in N protein fluorescence in compound **7**-treated hamsters, confirming the compound's potent antiviral activity in lowering pulmonary viral replication. Cell nuclei are counterstained with DAPI (blue). Images are representative of n = 6 animals per group.

**Figure S4. The fold changes of F/R after treatment with drugs in SARS and MERS.**


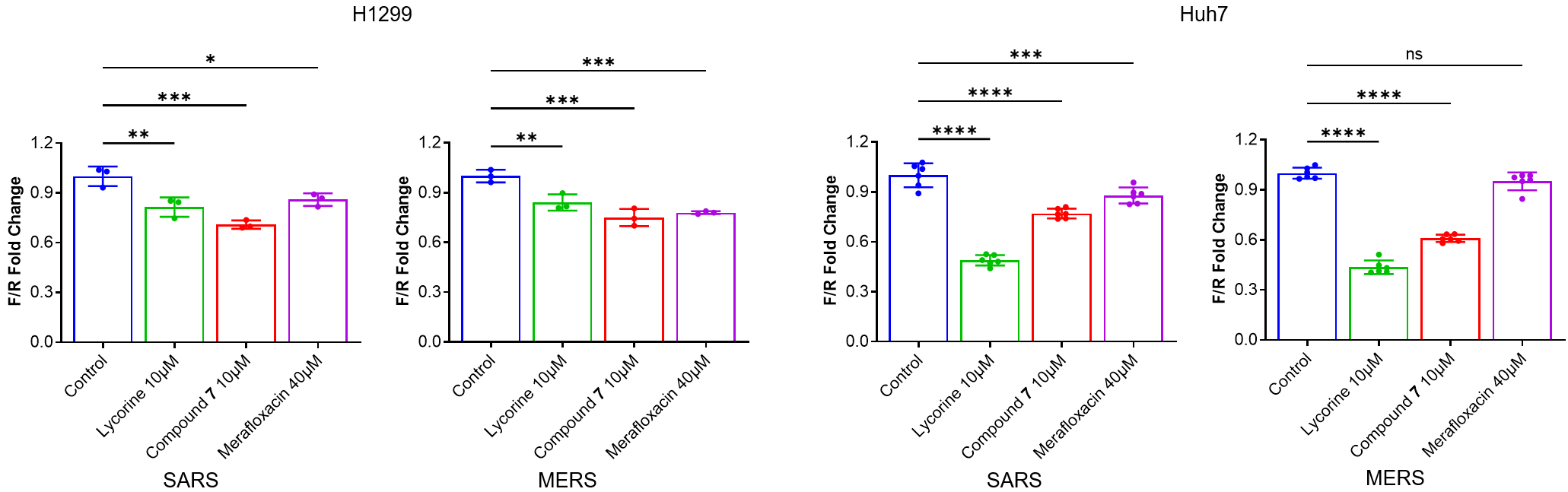


The fold changes of F/R after treatment with drugs in SARS and MERS. Huh-7 and H1299 cells transfected with the pHRF-FSE (–1) luciferase reporter vector of SARS-CoV-1 and MERS-CoV were treated with the DMSO (Control) or drugs for 48 hours. Lycorine (10 μM), compound 7 (10 μM), Merafloxacin (40 μM); n = 3 or 6 per group.

**Figure S5. Molecular docking results for ZAP with lycorine.**


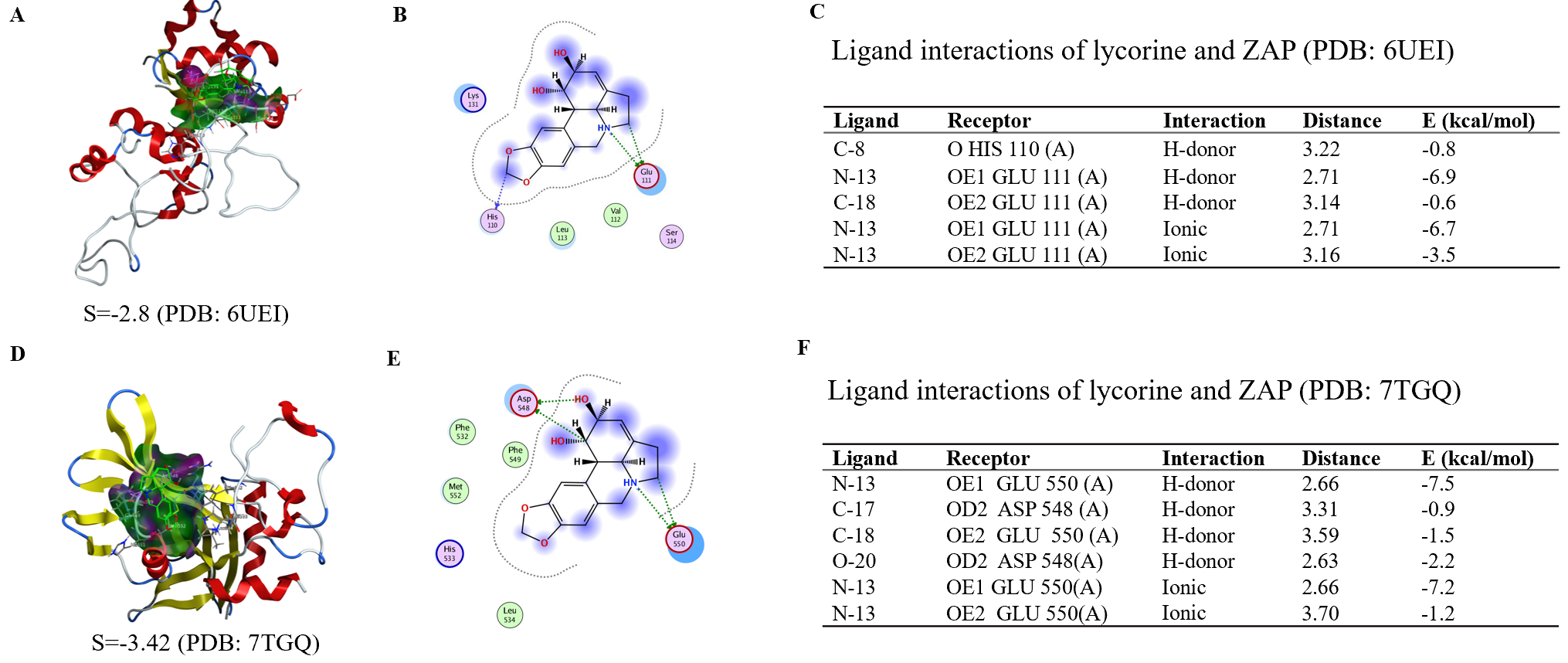


**A**. Molecular docking results for N-terminal residues of ZAP (PDB code: 6UEI) with lycorine. **B**. The 2D image reveals that compound **7** interacts with several amino acids His110 and Glu111. **C**. Atomic distance and binding energy of ZAP (PDB code: 6UEI) with lycorine molecular interaction. **D**. Molecular docking results for central domain of ZAP (PDB code: 7TGQ) with lycorine. **E**. The 2D image reveals that compound **7** interacts with amino acids Asp548 and Glu550. **F**. Atomic distance and binding energy of ZAP (PDB code: 7TGQ) with lycorine molecular interaction.

**Table S1.** **The CC_50_ values of lycorine and compounds 7 in Huh-7, H1299 and Cp-H209**

|  | Lycorine (CC_50_, μM) | Compound **7** (CC_50_, μM) |
| --- | --- | --- |
| Huh-7 | 46.86 ± 4.04 | 68.37 ± 8.45 |
| H1299 | 50.28 ± 1.64 | 72.46 ± 1.83 |
| CP-H209 | 38.73 ± 3.49 | 56.87 ± 3.49 |

**Table S3. The compound 7 potential targets identified by LC/MS-MS.**

| Gene name | MolWeight (kD) | FC | Function |
| --- | --- | --- | --- |
| CHERP | 103.7 | 2.05 | Enables transmembrane transporter binding activity. Involved in positive regulation of calcineurin-NFAT signaling cascade and release of sequestered calcium ion into cytosol. Acts upstream of or within intracellular calcium ion homeostasis and negative regulation of cell population proliferation. |
| FAM50A | 40.2 | 2.33 | It is a basic protein containing a nuclear localization signal, and may function as a DNA-binding protein or a transcriptional factor. |
| TRIM56 | 81.48 | 2.09 | Promotes establishment of an antiviral state by TLR3 ligand and TLR3 mediated chemokine induction following infection by hepatitis C virus. |
| ZAP | 101.43 | 2.06 | Antiviral protein which inhibits the replication of viruses by recruiting the cellular RNA degradation machineries to degrade the viral mRNAs. |
| RPS29 | 6.67 | 3.53 | This gene encodes a ribosomal protein that is a component of the 40S subunit and a member of the S14P family of ribosomal proteins. The protein, which contains a C2-C2 zinc finger-like domain that can bind to zinc, can enhance the tumor suppressor activity of Ras-related protein 1A (KREV1). |

**Table S4. Atomic distance and binding energy of molecular interaction.**

Ligand interactions of compound **7** and ZAP (PDB: 6UEI)

| Ligand | Receptor | Interaction | Distance | E (kcal/mol) |
| --- | --- | --- | --- | --- |
| N-13 | OE2 GLU 115 (A) | H-donor | 2.63 | -5.0 |
| C-17 | OE1 GLU 111 (A) | H-donor | 3.17 | -1.6 |
| O-20 | OE1 GLU 111 (A) | H-donor | 2.70 | -4 |
| N-13 | OE2 GLU 115 (A) | Ionic | 2.63 | -7.5 |

Ligand interactions of compound **7** and ZAP (PDB: 7TGQ)

| Ligand | Receptor | Interaction | Distance | E (kcal/mol) |
| --- | --- | --- | --- | --- |
| C-10  N-13 | OE2 GLU 550 (A)  OE1 GLU 550 (A) | H-donor  H-donor | 3.70  2.59 | -0.7  -15.2 |
| O-20 | O PHE 549 (A) | H-donor | 3.06 | -1.4 |
| N-13 | OE1 GLU 550(A) | Ionic | 2.59 | -7.9 |
| N-13 | OE1 GLU 550(A) | Ionic | 3.91 | -0.7 |

**Table S5.** **Primers used in this study.**

| Gene | Primer F | Primer R |
| --- | --- | --- |
| *NFKBIA* | CTCCGAGACTTTCGAGGAAATAC | GCCATTGTAGTTGGTAGCCTTCA |
| *RELB* | CCATTGAGCGGAAGATTCAACT | CTGCTGGTCCCGATATGAGG |
| *TNF* | CCTCTCTCTAATCAGCCCTCTG | GAGGACCTGGGAGTAGATGAG |
| *TNFAIP3* | TCCTCAGGCTTTGTATTTGAGC | TGTGTATCGGTGCATGGTTTTA |
| *MAP3K8* | CTCCCCAAAATGGACGTTACC | GGATTTCCACATCAGATGGCTTA |
| *SELE* | CAGCAAAGGTACACACACCTG | CAGACCCACACATTGTTGACTT |
| *ACTIN* | CATGTACGTTGCTATCCAGGC | CTCCTTAATGTCACGCACGAT |
| *NP* | GACCCCAAAATCAGCGAAAT | TCTGGTTACTGCCAGTTGAATCTG |
| *RdRp* | GGTAACTGGTATGATTTCG | CTGGTCAAGGTTAATATAGG |
| *Actin* | ATGGCCAGGTCATCACCATTG | CAGGAAGGAAGGCTGGAAAAG |

**Table S6. Sequences of primers used for plasmid constructions.**

| **plasmid** | **primers** |
| --- | --- |
| ZAP  (WT) | FORWARD:GGGGTACCCCGCCACCATGGACTACAAAGACCATGACGGTGATTATAAAGATCATGACATCGACTACAAGGATGACGATGACAAGATGGCGGACCCGGAGGTG  REVERSE: CGGAATTCCGTTACTCTGGCCCTCTCTTCATCTG |
| ZAP  (E111A) | FORWARD: TTCTCATgcgGTTCTCTCAGAAGAGAACTTCAAAGTCC  REVERSE: AGAGAACcgcATGAGAATATTTGCATAAATTCCGC |
| ZAP  (E115A) | FORWARD: TCTCTCAGcAGAGAACTTCAAAGTCCTGAAAAATCA  REVERSE: AGTTCTCTgCTGAGAGAACCTCATGAGAATATTTGC |
| ZAP  (F549A) | FORWARD: ACGGACgcgGAGCACATGGAGACGATCGAGAA  REVERSE: ATGTGCTCcgcGTCCGTCCAGGTTTTACCAATAA |
| ZAP  (E550A) | FORWARD: GACTTTgcgCACATGGAGACGATCGAGAAAGG  REVERSE: TCCATGTGcgcAAAGTCCGTCCAGGTTTTACCA |
